# Discrete Levels of Twist Activity Are Required to Direct Distinct Cell Functions during Gastrulation and Somatic Myogenesis

**DOI:** 10.1371/journal.pone.0099553

**Published:** 2014-06-10

**Authors:** Ming-Ching Wong, Krista C. Dobi, Mary K. Baylies

**Affiliations:** 1 Program in Developmental Biology, Sloan-Kettering Institute, New York, New York, United States of America; 2 Weill Graduate School at Cornell Medical School, New York, New York, United States of America; Institute of Molecular and Cell Biology, Singapore

## Abstract

Twist (Twi), a conserved basic helix-loop-helix transcriptional regulator, directs the epithelial-to-mesenchymal transition (EMT), and regulates changes in cell fate, cell polarity, cell division and cell migration in organisms from flies to humans. Analogous to its role in EMT, Twist has been implicated in metastasis in numerous cancer types, including breast, pancreatic and prostate. In the *Drosophila* embryo, Twist is essential for discrete events in gastrulation and mesodermal patterning. In this study, we derive a *twi* allelic series by examining the various cellular events required for gastrulation in *Drosophila*. By genetically manipulating the levels of Twi activity during gastrulation, we find that coordination of cell division is the most sensitive cellular event, whereas changes in cell shape are the least sensitive. Strikingly, we show that by increasing levels of Snail expression in a severe *twi* hypomorphic allelic background, but not a *twi* null background, we can reconstitute gastrulation and produce viable adult flies. Our results demonstrate that the level of Twi activity determines whether the cellular events of ventral furrow formation, EMT, cell division and mesodermal migration occur.

## Introduction

During development, cells are required to proliferate, differentiate and migrate at precise moments to achieve a functional organ or organism. Regulation of gene expression at the level of transcription has proven to be a key mechanism to coordinate such cellular functions [1]-[3]; however, it remains an open question how a single transcription factor can coordinate multiple cellular events. The conserved basic helix-loop-helix transcriptional regulator Twist (Twi) is a transcription factor with multiple roles within one tissue throughout development [4]. Early in *Drosophila* development, Twi is essential for gastrulation, the process that forms the mesoderm. Twi later acts to specify and pattern the somatic mesoderm. Subsequently, Twi expression in adult muscle progenitors is required to regulate proliferation and maintain pluripotency until the onset of adult myogenesis [5]. Integral to its role in these processes, Twi activity is required for myriad discrete cell behaviors, but the mechanisms by which Twi exerts this pleiotropic control are unclear.

For gastrulation to occur, mesodermal cells must be specified, change cell shape to form the ventral furrow (VF), divide synchronously, undergo an epithelial-to-mesenchymal transition (EMT) and, finally, migrate along the ectoderm to form a layer of mesodermal cells. The specification of the mesodermal primordium relies on the expression of *twi,* and these discrete cellular events are either missing or severely impaired in *twi* mutant embryos [6], [7]. At gastrulation, an 18–20 cell diameter of the ventral most cells of the blastoderm express *twi* [8]. *twi* expression is activated by high nuclear levels of Dorsal (Dl), the *Drosophila* homolog of the transcriptional regulator, NF-KB [6]. High levels of nuclear Dl are required for the zygotic expression of both *twi* and *snail* (*sna*), genes that confer mesodermal cell fates [9].

The combinatorial activities of Twi and Sna promote mesodermal specification. Sna is a zinc-finger transcriptional regulator that is required for the cell shape changes that are necessary for VF formation and gastrulation [10], [11]. Although Sna expression is first activated by Dl, Twi is required to maintain Sna expression at high levels [9]. It is apparent that one way Twi can coordinate the disparate processes of gastrulation is through a feed-forward mechanism. Twi activates target genes that then work in concert with Twi itself to activate different subsets of targets. For example, while Dl initially activates Sna, Sna expression requires Twi to maintain its expression. Once Sna is activated, both Twi and Sna are required to maintain Twi expression as well as another set of target genes. This mechanism is integral in coordinating the activities of Dl, Twi and Sna [7], [8], [12]–[14].

Twi and Sna work in concert to regulate the expression of distinct groups of mesodermal genes. Twi and Sna activate mesodermal transcription factors, such as *tinman (tin)*, *heartless* (*htl*) and *Drosophila myocyte enhancing factor 2* (*Dmef2*) [15]–[25]. Additionally, Twi activates genes required for apical constriction and invagination of the mesodermal cells, such as *Folded gastrulation, T48* and *Traf4* [26]–[28]. Sna was initially identified as a transcriptional repressor, and Sna represses non-mesodermal genes that are required for mesectoderm development, such as *rhomboid* (*rho*) and *single-minded* (*sim*) [7], [29].

Once specified, mesodermal cells undergo a series of morphogenic movements to create a ventral furrow (VF). First, the Twi-expressing cells flatten apically, elongate along their apical-basal axis and constrict at the apical membrane to form wedge-shaped cells [30]. These changes cause an indentation, driving the invagination of the mesodermal primodium, which eventually becomes completely internalized to form the VF and, later, the mesodermal anlagen [30], [31]. Upon the formation of the VF, the invaginated mesodermal cells undergo EMT, losing their close contact with neighboring cells and resulting in VF collapse. Mesodermal cells next migrate dorsally, then establish contact with the ectodermal cells and exhibit a reduction in the adhesive molecule Shotgun/DE-cadherin (Shg) expression [32].

Twi also regulates the cell cycle during gastrulation. Prior to invagination, cell division is arrested in mesodermal primordial cells. When these cells have invaginated and acquire mesenchymal characteristics, they are released from cell cycle arrest and undergo a wave of cell division [33]. This coordination of cell division is also carefully orchestrated by Twi, which both negatively and positively regulates *string/cdc25* activity [34], [35].

Whole genome chromatin immunoprecipitation studies have identified a large number of mesodermal genes directly regulated by Twi [13], [36]; however, our knowledge of how Twi activity is regulated at these target genes is incomplete. Twi proteins bind as dimers to E-box sequences with the consensus sequence 5′ CANNTG 3′ [17], [37], [38]. This dimerization plays a significant role in the regulation of Twi activity. Twi forms a homodimer that activates transcription of somatic mesoderm gene targets [39]. In flies as well as vertebrates, Twi also forms heterodimers with other bHLH proteins that modulate its activity. In *Drosophila*, Twi heterodimerizes with the E-box transcription factor Daughterless, and this heterodimer represses gene targets in the somatic mesoderm [39], [40]. Little is known about the other factors Twi recruits to target genes to mediate activation or repression. In adult muscle progenitors, Twi recruits Suppressor of Hairless to target genes, thereby integrating Twi activity with Notch signaling in these cells [41]. Recent work provides evidence that, in the somatic mesoderm, Twi recruits the Brahma chromatin remodeling complex to target genes via the novel cofactor Akirin [42]. The full picture of how Twi integrates cellular signals with chromatin remodeling and recruitment of the general transcription machinery remains to be elucidated. Throughout our work we refer to the sum of these Twi interactions, including both those that have been described and those functions that are as yet uncharacterized, as Twi activity. The level of Twi activity required at specific target genes may be different in subsets of tissues or at different times in development, and depend upon the specific function being performed by Twi at that target gene.

While Twi is essential for the coordination of the cellular events required for gastrulation, including cell specification, cell cycle regulation, EMT, and cell migration, it is unclear how Twi modulates the expression of target genes and the cellular events that rely on these genes. We took a genetic approach to analyze the effects of Twi on target genes and on the cellular events necessary for gastrulation and mesodermal migration (VF formation, EMT, cell division, and migration). Our analyses show that coordinated cell division can be perturbed in the weakest allelic background, while the process of cell shape change is altered in only the strongest allelic combination. Moreover, we show that gastrulation and lethality in a *twi* hypomorphic background can be rescued when Sna expression is artificially extended. This work sheds light on how one transcription factor can be used repeatedly in development to activate sets of target genes and direct diverse cellular functions. Additionally, our results enrich our understanding of Twi as a regulator of gastrulation, the cell cycle and the EMT, knowledge that will aid in the understanding of Twi as a regulator of cell fate and behavior during mammalian development and disease.

## Results

### The Establishment Of A *Twi* Allelic Series

We took a genetic approach to manipulate Twi *in vivo* using combinations of null and hypomorphic alleles of *twi* [6], [9]. The null allele, *twi^1^* produces *twi* mRNA, but not protein [43]. The hypomorphic alleles, *twi^V50^* and *twi^RY50^*, are both point mutations which produce full length protein. These *twi* alleles are embryonic homozygous lethal and have been shown to have different effects on mesoderm differentiation. Heterozygotes for these alleles appear wild-type [6], [9], [31]. To establish an allelic series, we examined embryos with each combination of these alleles for mutant phenotypes. In combination with the *twi^1^* null allele, the *twi^V50^* and *twi^RY50^* hypomorphic alleles gave rise to a variety of allelic combinations *in vivo* (ex. *twi^V50^*/*twi^V50^*, *twi^V50^*/*twi^1^, twi^RY50^/twi^RY50^, twi^RY50^*/*twi^1^*, and *twi^1^*/*twi^1^*). This series allowed us to investigate gastrulation and mesoderm formation in allelic combinations of varying severity. Because mesoderm patterning and somatic myogenesis are sensitive to the level of Twi activity [40], the final somatic muscle pattern of the allelic combinations was first analyzed.

**Figure 1:**
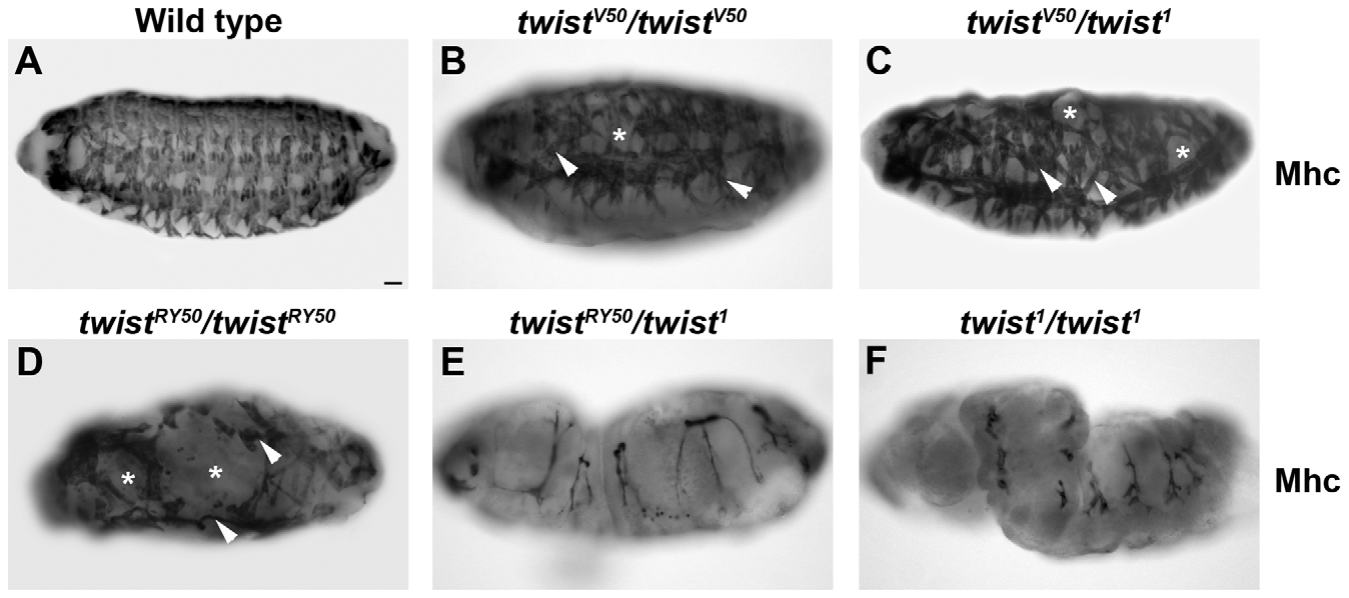
Establishment of the Twi allelic series. (A–F) Lateral views of whole mount stage 16 embryos stained with anti-Myosin heavy chain (Mhc). The final muscle pattern of wild-type embryos (A), *twi^V50^/twi^V50^* (B), *twi^V50^/twi^1^* (C), *twi^RY50^/twi^RY50^* (D), *twi^RY50^/twi^1^* (E), and *twi^1^/twi^1^* (F) are shown. Arrowheads indicate incorrect muscle morphologies and muscle losses are noted by asterisks. Scale bar, 20 µm.

When stained for Myosin heavy chain (Mhc), wild-type embryos displayed a segmentally repeated, stereotypic pattern of 30 somatic muscles per abdominal hemisegment (Figure 1A). *twi^V50^*/*twi^V50^* embryos displayed muscle loss and minor muscle patterning defects (Figure 1B). *twi^V50^*/*twi^1^* embryos exhibited more severe muscle loss and muscle patterning defects (Figure 1C). *twi^RY50^/twi^RY50^* embryos were missing most of the somatic muscles, and those that form were severely mispatterned (Figure 1D). *twi^RY50^*/*twi^1^* embryos did not form muscle at all (Figure 1E) and had a phenotype similar to homozygous null embryos, *twi^1^*/*twi^1^* (Figure 1F). The final muscle pattern of the various allelic combinations revealed a clear allelic series, reflecting different levels of Twi activity. These results support previously published results that also described the *twi^RY50^* allele as a stronger hypomorph than *twi^V50^* [6], [9].

### Twi Target Genes Have Different Activation Thresholds

We next examined expression of several well-characterized Twi target genes to assess whether the different *twi* allelic combinations could activate gene expression at multiple stages in development. Specifically, we characterized the expression of Sna (stages 5 and 7), Htl (stage 8) and Dmef2 (stage 10) to assess Twi activation of target genes during gastrulation, mesodermal migration and myogenesis.

**Figure 2:**
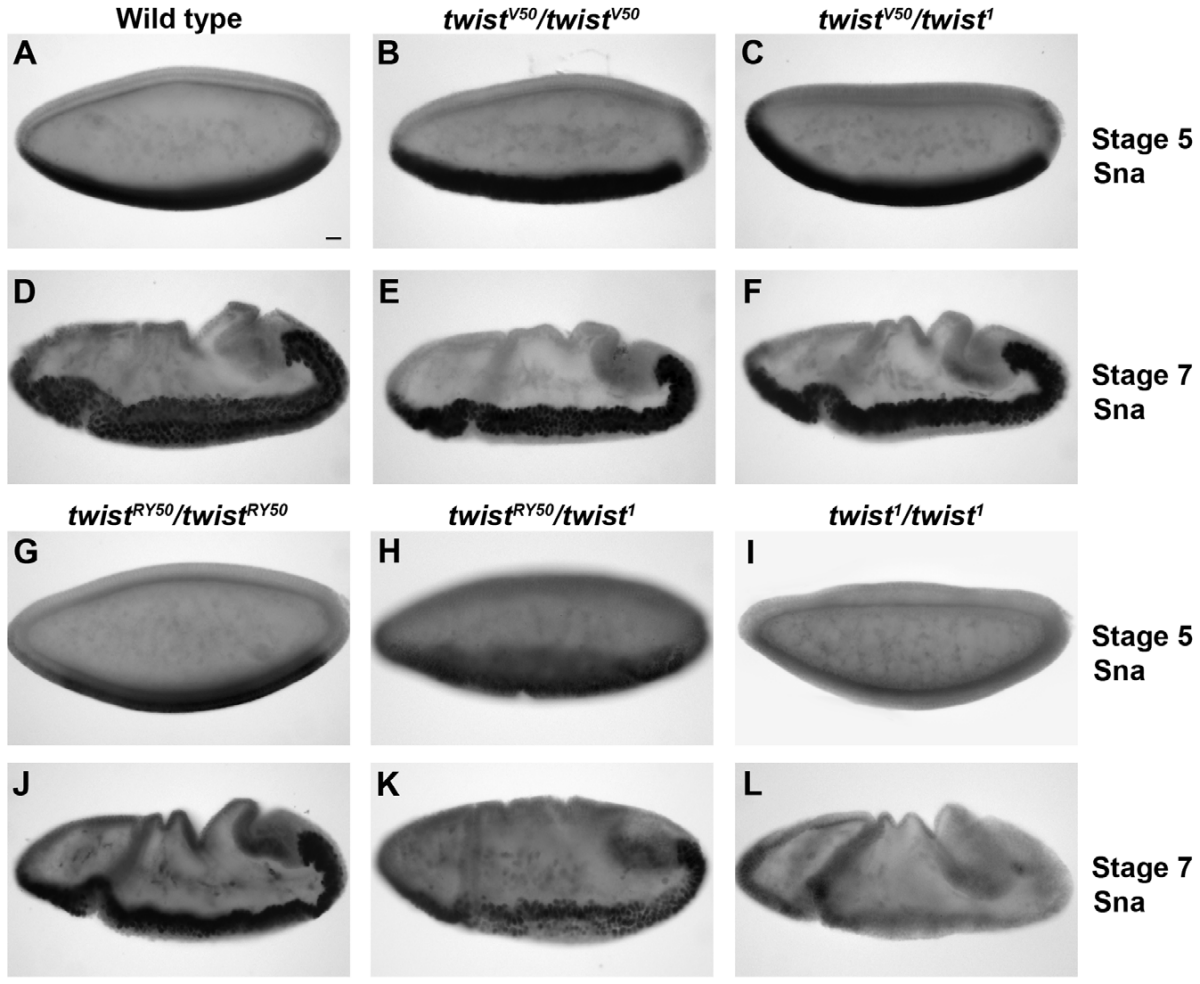
Sna expression in increased in *twi* mutant embryos. Lateral views of wild-type (A,D), *twi^V50^/twi^V50^* (B,E), *twi^V50^/twi^1^* (C,F), *twi^RY50^/twi^RY50^* (G,J), *twi^RY50^/twi^1^* (H,K), and *twi^1^/twi^1^* (I,L) embryos at stage 5 (A–C, G–I) and stage 7 (D–F, J–L) stained with anti-Sna antibody. Scale bar, 20 µm.

Sna expression is strongly activated and maintained by Twi in the ventral most cells in the blastoderm in wild-type embryos (Figure 2A). This mesodermal expression is maintained through gastrulation (Figure 2D). In *twi^1^/twi^1^* null embryos, only a faint level of Sna can be detected at stage 5, and no Sna is detected at stage 7 (Figure 2I, L). The low level visible at stage 5 is attributed to the independent activating effect of Dorsal [28]. We next examined the other allelic combinations. Sna expression can be detected in *twi^V50^/twi^V50^* (Figure 2B,E) and *twi^V50^/twi^1^* (Figure 2C,F) embryos as in wild-type embryos. *twi^RY50^/twi^RY50^* embryos, however, have reduced Sna expression at stage 5 (Figure 2G). Despite this reduction at stage 5, stage 7 *twi^RY50^/twi^RY50^* embryos expressed Sna at levels comparable to wild-type (Figure 2J), which indicated that low levels of Twi activity can lead to the accumulation of wild-type Sna levels over time. In contrast, Sna levels are reduced in *twi^RY50^/twi^1^* embryos, both at stage 5 (Figure 2H) and at stage 7 (Figure 2K). Ventral views of these embryos at stage 5 revealed that the number of cells expressing Sna was also reduced (Figure S1). Hence, in stronger allelic combinations, the embryo was unable to activate appropriate Sna levels, regardless of the passage of developmental time. Taken together, these results suggested that Sna levels are directly related to Twi activity. Moreover, even embryos with severe *twi* allelic combinations were able to activate Sna expression *in vivo*.

**Figure 3:**
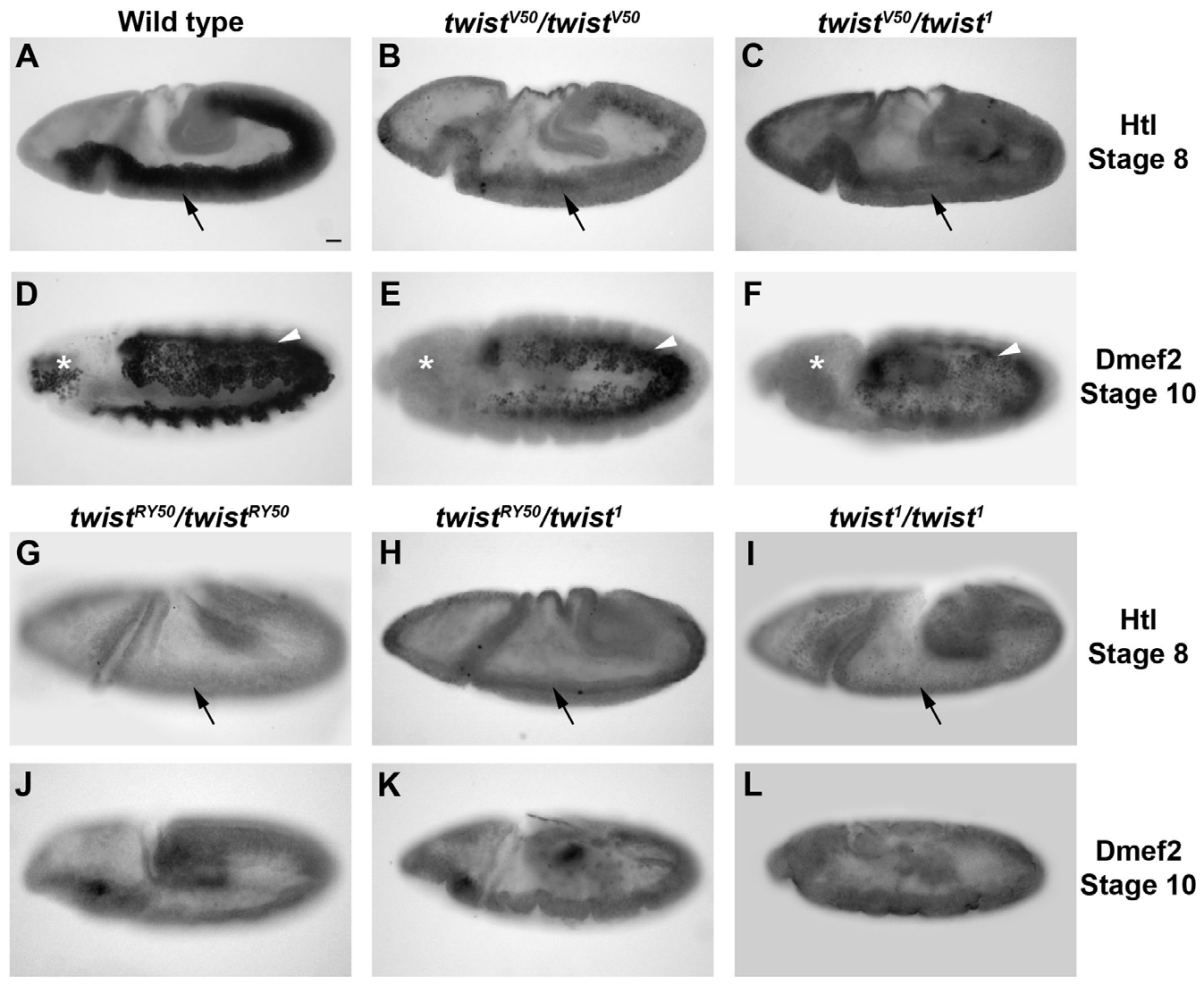
Htl and Dmef2 expression is sensitive to the *twi* allelic combination. Lateral views of wild-type (A,D), *twi^V50^/twi^V50^* (B,E), *twi^V50^/twi^1^* (C,F), *twi^RY50^/twi^RY50^* (G,J), *twi^RY50^/twi^1^* (H,K), and *twi^1^/twi^1^* (I,L) embryos are shown. Embryos at stage 8 stained with anti-Htl (A–C, G–I), and embryos at stage 10 stained with anti-Dmef2 antibody (D–F, J–L) are shown. Black arrows indicate ventral cells that should express Htl. White arrowheads indicate mesodermal cells that express Dmef2 and have migrated. White asterisks indicate the position of pharyngeal progenitor cells, which are not visible in mutants. Scale bar 20 µm.

Expression of Heartless (Htl), a fibroblast growth factor receptor, is required for the initial phase of mesodermal migration that takes place upon the completion of gastrulation [44]. We evaluated Htl levels in embryos carrying different Twi allelic combinations. In stage 8 wild-type embryos, Htl was highly expressed in mesodermal cells (Figure 3A). In contrast, Htl was only weakly expressed in *twi^V50^/twi^V50^* and *twi^V50^/twi^1^* embryos (Figure 3B,C) and cannot be detected in the mesoderm of *twi^RY50^/twi^RY50^, twi^RY50^/twi^1^*, and *twi^1^/twi^1^* (Figure 3G,H,I). These results indicated that Htl expression was more sensitive to Twi activity levels than Sna, since Htl expression is reduced even in weak *twi* allelic combinations. Also, the very low or loss of Htl expression in *twi^RY50^/twi^RY50^* and *twi^RY50^/twi^1^* embryos suggested that these embryos may exhibit defects in mesodermal migration. We therefore examined a marker of later mesodermal development.

*Drosophila* myocyte enhancing factor 2 (Dmef2) is a transcriptional regulator required for all muscle development [17]. The pattern of Dmef2 staining was used to determine whether mesodermal migration was affected in *twi* mutants. In wild-type embryos, Dmef2 was expressed uniformly within the mesoderm at stage 10 (Figure 3D). Overall, Dmef2 levels were reduced in *twi^V50^/twi^V50^* embryos, but these Dmef2-positive cells migrated dorsally and exhibited proper patterning similar to wildtype, with the exception of the pharyngeal muscle progenitors, which failed to express Dmef2 (Figure 3E). *twi^V50^/twi^1^* embryos expressed Dmef2 at a much reduced level of expression, and pharyngeal muscle progenitors could also not be detected (Figure 3F). Although the Dmef2-expressing cells appeared to migrate dorsally, these cells were not patterned properly and, unlike wild-type embryos, these expressed lower levels of Dmef2. Finally, *twi^RY50^/twi^RY50^, twi^RY50^/twi^1^*, and *twi^1^/twi^1^* embryos had undetectable levels of Dmef2, which suggested that mesodermal cells, if any are specified, were not achieving the correct cell fates (Figure 3J,K,L). These data demonstrated that Dmef2 expression was also sensitive to Twi activity levels, its response similar to what was seen with Htl.

Taken together, these results suggested that different target genes have different requirements for Twi. For example, Sna can be detected in *twi^RY50^/twi^1^* embryos with very low Twi activity, while Htl cannot be detected. These results further supported the idea that the specificity of transcriptional regulation is particular to each target gene, and that even a common transcriptional regulator, in this case Twi, is not sufficient to predict target gene output.

### *twi* Mutant Embryos Display Reduced Ventral Furrow Size And Mesodermal Cells

Preliminary observations from *twi* mutant embryos revealed defects to processes essential for mesoderm formation, such as cell shape changes required for VF formation and mesodermal cell migration. For example, Sna staining revealed that *twi^RY50^/twi^1^* embryos exhibit disrupted VF formation (Figure 2K), and Dmef2 staining revealed disorganized cell migration in *twi^V50^/twi^1^*embryos (Figure 3F). To better analyze the cellular processes that are required for gastrulation and mesodermal migration, transverse sections of gastrulating embryos were stained for Shotgun (DE-cadherin) and phalloidin to visualize F-actin.

**Figure 4:**
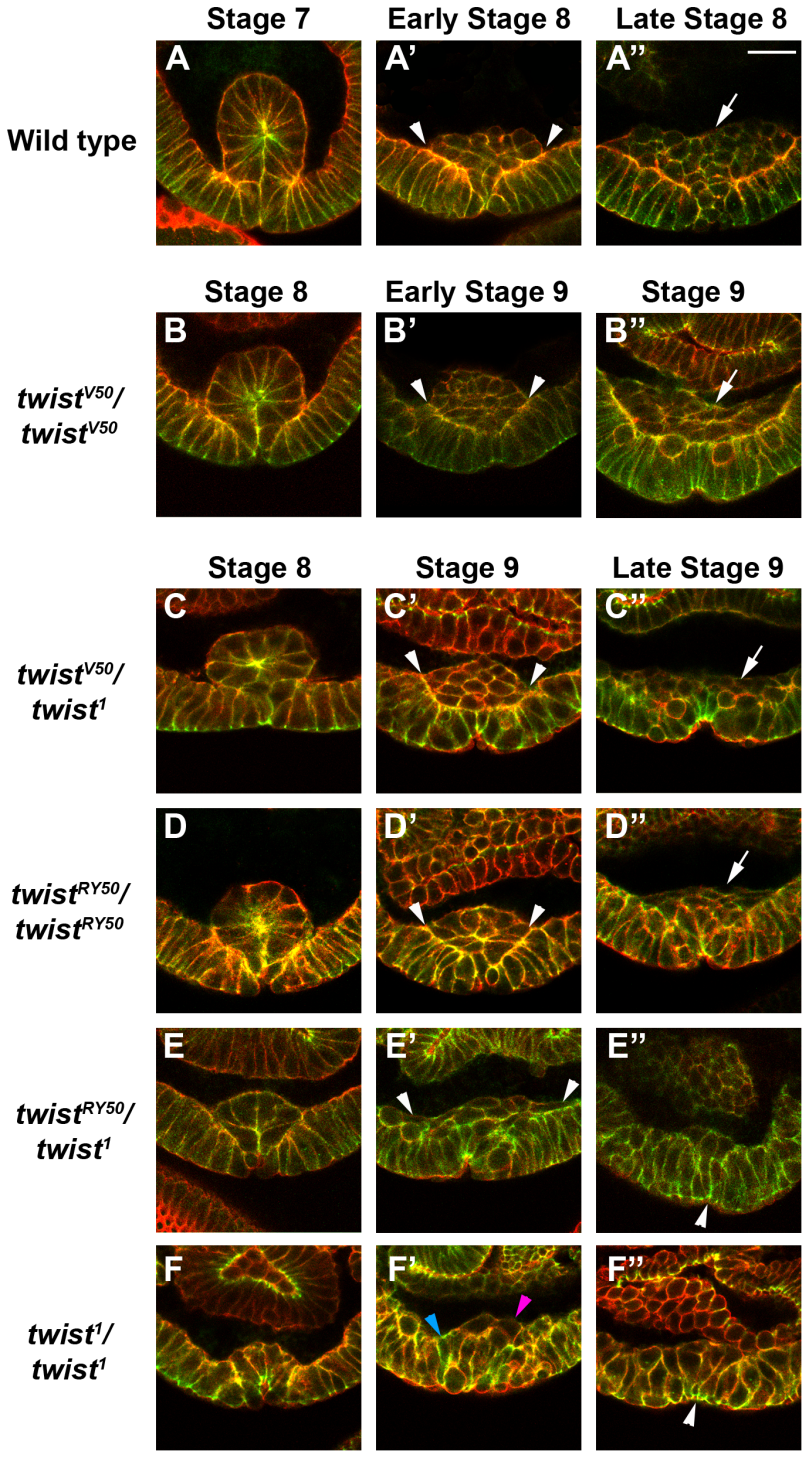
Ventral furrow formation, epithelial to mesenchymal transition (EMT), cell division and migration respond to Twi activity levels. Confocal micrographs of transverse sections of *wild-type* (A–A″), *twi^V50^/twi^V50^* (B–B″), *twi^V50^/twi^1^* (C–C″), *twi^RY50^/twi^RY50^* (D–D″), *twi^RY50^/twi^1^* (E–E″), and *twi^1^/twi^1^* (F–F″) embryos stained with phalloidin to visualize F-actin (red) and an antibody raised against Shotgun (green); colocalization of F-actin and Shotgun appears yellow. Scale bar, 20 µm. (A–F) At stage 7 (*wild-type*) or stage 8, Phalloidin and Shotgun expression reveal cell shapes and Shotgun expression is enriched at sites of apical constriction. (A′–F′) Shotgun expression is higher in the ectodermal cells than in the mesodermal cells undergoing the EMT. Mesodermal cells that have contacted the ectodermal cells are indicated by arrowheads (A′–E′). *twi^1^/twi^1^* embryos have few mesenchymal cells (pink arrowhead); most retain an ectodermal morphology (blue arrowhead). The wild-type embryo is at stage 8, the *twi^V50^/twi^V50^* embryo is at early stage 9, and all other embryos are at stage 9. (A″–F″) White arrows indicate mesodermal cells that have divided and started the first phase of migration (A″, B″, C″, D″). *twi^RY50^/twi^1^* (E″) and *twi^1^/twi^1^* (F″) embryos do not form mesodermal cells that divide or migrate; instead, cells return to the ectodermal layer of cells and appear to reverse the cell shape changes that give rise to the furrow (white arrowheads). The wild-type embryo shown is at late stage 8, the *twi^V50^/twi^V50^* embryo is at stage 9, and all other embryos are at late stage 9.

By late stage 7 in wild-type embryos, mesodermal cells were fully invaginated via the VF (Figures 4A, S2A, S3A). These invaginated cells underwent EMT by mid stage 8, followed by a round of cell division. Subsequently, these cells migrated away from the ventral midline along the ectoderm by early stage 9 (Figures 4A′–A″, S2C, S2E, S4A, S5A). In contrast, *twi^1^/twi^1^* null embryos were developmentally delayed, as previously published [6]. Late stage 7 *twi^1^/twi^1^* embryos exhibited little to no ventral cell shape changes (Figure S2B), and the closest semblance to the formation of a VF occurred by stage 8 (Figures 4F, S2D, S3F). By early stage 9, the cell shape changes had become disorganized, and the cells that formed a minor VF were unable to maintain the shape changes; therefore cell invagination was lost (Figures 4F′, S2F, S4F).

While *twi^1^/twi^1^* embryos had the most extreme phenotype, analysis of the other *twi* allelic combinations revealed a developmental delay that correlated with the *twi* allelic series (Table S1). Notably, this developmental delay had its own threshold response to Twi: *twi^V50^*/*twi^V50^* embryos, with the exception of VF formation, exhibited an overall developmental delay of 5 minutes, whereas the other allelic combinations exhibited a 15 to 20 minute delay (Table S1). These results suggested that embryos with reduced Twi activity levels required more time to accomplish the cell processes necessary to complete gastrulation.

Next, the cell shape changes required to form a VF were analyzed. To compare the cell shape changes between embryos of different genetic backgrounds, we compared embryos at the point at which the VF was at its greatest degree of formation for each genetic background instead of developmental staging or time. With this approach, the maximal changes in cell shape could be analyzed and developmental delay would not affect VF analysis.

Phalloidin and Shotgun staining revealed that the ventral cells of *twi^V50^*/*twi^V50^*, *twi^V50^*/*twi^1^*, and *twi^RY50^*/*twi^RY50^* embryos underwent cell shape changes to give rise to a robust VF structure (Figure 4A–D, S3A–D″). Interestingly, different numbers of cells formed the VF in each allelic combination. For example, the VF in wild-type embryos consisted of 15 to 18 cells, with an average number of 16 cells (Figure 4A, S3A–A″; Table S2) [7]. By comparison, *twi^V50^*/*twi^V50^* embryos, which carry the weakest hypomorphic allele combination of the series, formed VFs that were composed of 13 cells on average (Figure 4B, S3B–B″; Table S2). Following this trend, an average of 11 cells formed the VF in *twi^V50^*/*twi^1^* embryos (Figure 4C, S3C–C″), and *twi^RY50^*/*twi^RY50^* embryo VFs were composed of an average of 9 cells (Figure 4D, S3D–D″; Table S2).

In contrast, *twi^RY50^*/*twi^1^* and *twi^1^*/*twi^1^* embryos exhibited a similar number of ventral cells (an average of 7 cells) and cell shape changes to those cells, but were unable to form a proper VF structure (Figure 4E–F, S3E–F″; Table S2). In general, however, the 6 to 8 ventral cells in *twi^RY50^*/*twi^1^* embryos (Figure 4E, S3E–E′; Table S2) underwent more consistent cell shape changes and invagination that resulted in a structure that resembled the VF more than *twi^1^*/*twi^1^* null embryos (Figure 4F, S3F–F″). The cells that formed the VF in *twi^1^*/*twi^1^* embryos underwent inconsistent apical constriction and showed very little displacement from the ectoderm (Figure 4F, S3F–F″).

Taken together, these results confirmed that Twi is crucial for both the timing of VF formation and its structure. Notably, the results revealed that the severity of the *twi* allele determined the number of cells that invaginate to form the mesoderm.

### The Proper Level Of twi Activity Is Required For Mesodermal Cells To Undergo The Epithelial-To-Mesenchymal Transition, Cell Divisions And Cell Migration

In wild-type embryos, the cells of the VF undergo an EMT, proliferate, and migrate to form the mesoderm. We therefore asked whether the smaller VFs observed in the *twi* allelic series could perform these three functions. Normally, cells that make up the VF first go through an EMT before migrating. These cells lose their epithelial shape and characteristics, coinciding with the collapse of the VF, allowing mesodermal cells to make contact with the ectoderm (Figure 4A′, S4A). Once cells complete the EMT, a slight reduction of Shotgun expression is seen in mesodermal cells compared to ectodermal cells (Figure 4A′, S4A′–A″). In *twi^V50^*/*twi^V50^*, *twi^V50^*/*twi^1^*, and *twi^RY50^*/*twi^RY50^* embryos, the VF collapsed against the ectoderm and adopted a mesenchymal shape, similar to wild-type embryos (Figure 4B′–D′, S4B,C,D). The mesodermal cells in these embryos also displayed slightly reduced levels of Shotgun in comparison to the ectodermal cells in the same embryo (Figure 4 B′–D′, S4B′,B″,C′,C″,D′,D″). Although only a few cells went through the EMT in *twi^RY50^*/*twi^1^* embryos, these mesodermal cells exhibited mesenchymal morphology (Figure 4E′, S4E) and also expressed reduced Shotgun levels (Figure 4E′, S4E′–E″). Finally, the ventral cells of *twi^1^/twi^1^* null embryos were unable to undergo the EMT. Phalloidin staining revealed that the ventral cells that had undergone apical constriction returned to a more columnar morphology and rejoined the ectodermal layer of cells (Figure 4F′, S4F). Moreover, these ventral-most cells expressed similar Shotgun levels compared to their adjacent ectodermal cells (Figure 4F′, S4F′–F″). These results demonstrated that initiation of the EMT could take place in even the most severe *twi* alleic combinations. In fact, any hypomorphic combination of Twi alleles, except for the *twi^1^/twi^1^* homozygous null embryos, was adequate to initiate the EMT.

To properly form the mesoderm after the EMT, mesodermal cells undergo a first round of synchronized mitosis and the first phase of mesodermal migration along the ectodermal cells. Mesodermal cells begin this phase of development as a mound of cells, then distribute themselves into a sheet of cells overlying the ectoderm. In wild-type embryos, the number of mesodermal cells increased during this distribution (compare Figure 4A′ to 4A″ and S4 A–A′″ to S5A–A″), which indicated that both proliferation and migration occurred. Similar to wild-type embryos, *twi^V50^*/*twi^V50^* embryos showed proliferation of mesodermal cells that spread along the ectodermal cells (Figure 4B″, S5B–B″). Interestingly, *twi^V50^*/*twi^1^* embryos also exhibited mesodermal cell spreading on the ectoderm (Figure 4C″, S5C′–C″), but there were fewer mesodermal cells than in wild-type or *twi^V50^*/*twi^V50^* embryos (compare Figure 4 C″ to 4A–B″, and S5C′–C″ to S5A–B″). In *twi^RY50^*/*twi^RY50^* embryos, mesodermal cells can spread, yet the lack of proliferation of mesodermal cells appeared even more pronounced in these embryos (Figure 4 D″, S5D–D″”).

Curiously, the mesodermal cells that underwent EMT in *twi^RY50^*/*twi^1^* embryos at earlier stages of development were no longer observable in these embryos at later stages of development (compare Figure 4 E′ to E″, and S4E–E″ to S5E–E″). This finding correlated with the observation that mesodermal cells cannot be identified at later stages of *twi^RY50^*/*twi^1^* embryo development using markers such as Dmef2 or Htl (Figure 3H,K). To determine whether these mesodermal cells had apoptosed, a cleaved caspase-3 antibody was used to detect the presence of apoptotic cells. Staining of *twi^RY50^*/*twi^1^* embryos with this antibody revealed no apoptotic mesodermal cells (data not shown). This result suggested two possibilities: the mesodermal cells in *twi^RY50^*/*twi^1^* embryos have undergone the reverse transition, the mesenchymal-to-epithelial transition (MET), to rejoin the ectodermal layer of cells, or have been unable to maintain a mesodermal cell fate and are no longer expressing mesodermal markers. Finally, *twi^1^*/*twi^1^* embryos did not form mesoderm, and any previously invaginated cells did not migrate and remained part of the ventral ectoderm (Figure 4F″, S5F-F″). Taken together, these data indicated that mesodermal migration, as well as the maintenance of the EMT or mesodermal fate, required a level of Twi activity that is not met by *twi^RY50^*/*twi^1^* or *twi^1^* homozygous embryos. The apparent reversal of EMT observed in *twi^RY50^*/*twi^1^* embryos suggested that a threshold level of Twi activity was required to maintain the changes in cell morphology and behavior characteristic of this transition.

**Figure 5:**
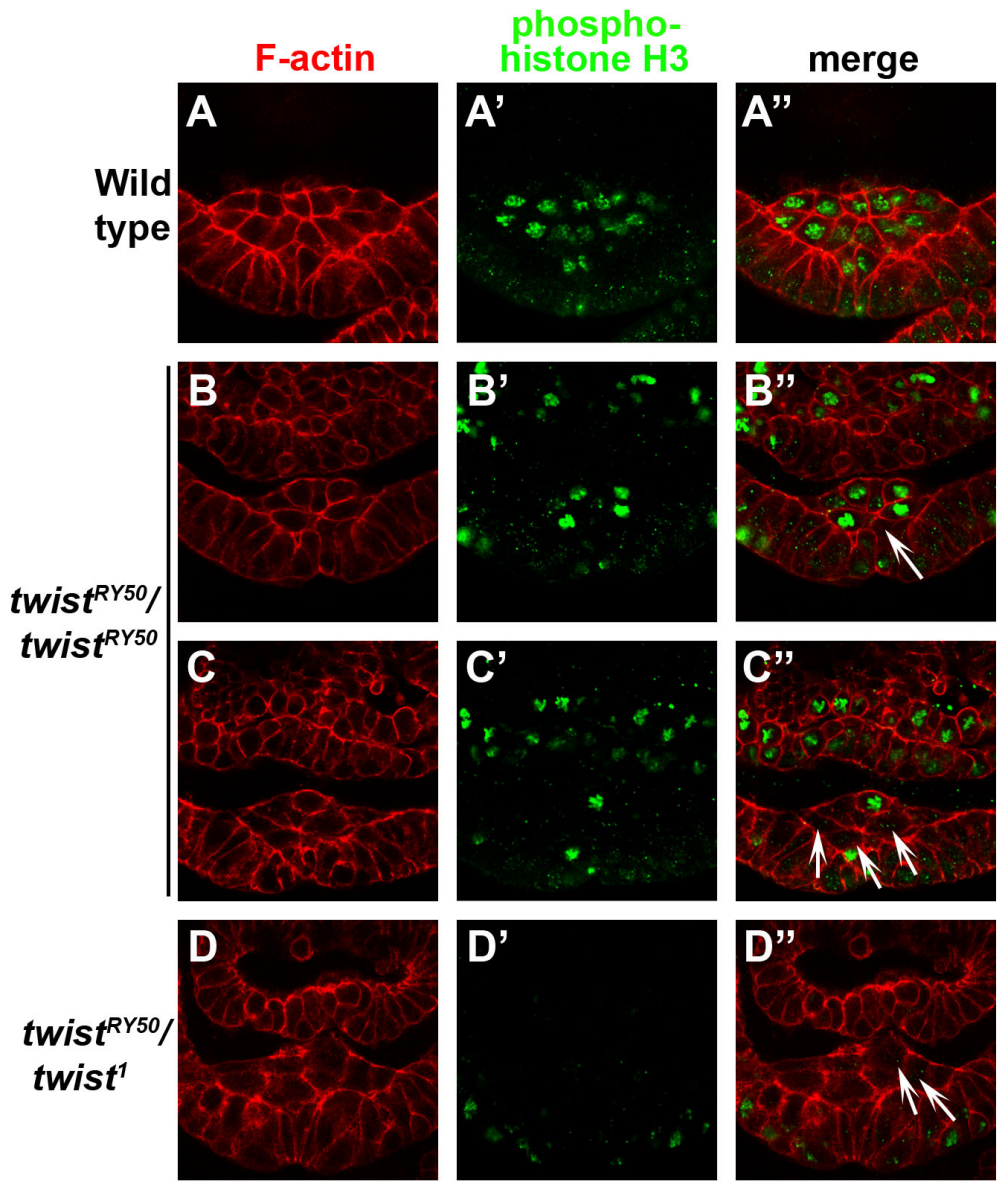
In strong *twi* allelic combinations, the first wave of mesodermal cell division becomes asynchronous and then ceases to occur. Transverse sections of control (A–A″), *twi^RY50^/twi^RY50^* (B–C″), and *twi^RY50^/twi^1^* (D–D″) embryos stained with phalloidin (red; A, B, C, D) and anti-PHH3 antibody (green; A′, B′, C′, D′). Merged panels are shown in A″, B″, C″, D″. White arrows indicate not dividing or asynchronously dividing mesoderm cells. The control embryo shown is at stage 8, and all other embryos are at stage 9. Scale bar, 20 µm.

Because we observed a reduced number of mesodermal cells in *twi^V50^*/*twi^1^, twi^RY50^*/*twi^RY50^*, and *twi^RY50^*/*twi^1^* embryos, we next determined whether this reduction was due to a reduced initial number of invaginated mesodermal cells or due to defects in cell division, using antibodies specific to the mitotic marker phospho-histone H3 (PHH3). In control embryos, the first wave of mitosis occurred synchronously in all mesodermal cells as they migrated along the ectoderm (Figure 5A–A″). Similar to the control, *twi^V50^*/*twi^1^* embryos also exhibited uniform PHH3 expression in mesodermal cells (data not shown). This result suggested that the first mesodermal mitotic wave proceeded normally and did not contribute to the reduced number of mesodermal cells in *twi^V50^*/*twi^1^* embryos (data not shown). Interestingly, *twi^RY50^*/*twi^RY50^* embryos underwent this first wave of mitosis (Figure 5B–B′), but not always in a synchronized manner (Figure 5C–C″). Finally, no PHH3 expression was detected in *twi^RY50^*/*twi^1^* embryos in the invaginated mesodermal cells (Figure 5D–D″), which indicated that mitosis did not occur in this cell population. These data demonstrated that disruption of mesodermal mitotic synchronicity was the process most easily perturbed in *twi* mutants, while stronger *twi* allelic combinations prevented mitosis altogether.

Furthermore, comparison of the different allelic combinations showed that there were several steps at which the final number mesodermal cells may be altered. For example, mitosis proceeded normally in *twi^V50^*/*twi^1^* embryos and the reduced number of mesodermal cells was due to a smaller VF in this allelic background. In contrast, mitosis occurred asynchronously in *twi^RY50^*/*twi^RY50^* embryos, which, in combination with a smaller VF, contributed to fewer mesodermal cells overall. Finally, mitosis was not detected in *twi^RY50^*/*twi^1^* mesodermal cells, and therefore the reduced mesodermal cells observed in these embryos derived only from the VF structure. Taken together, this analysis of mesodermal cell proliferation and migration reflected the close connection between Twi activity levels and cell processes required to form the mesoderm.

### Increasing Levels Of *twi* Disrupts Mesodermal Mitotic Synchrony

We next examined the effect of increased Twi levels on the multi-step processes of gastrulation and mesoderm formation using the Gal4-UAS system [45]. Though our loss-of-function data would lead us to predict that Twi overexpression in mesodermal cells would result in more rapid gastrulation and/or an increase in mesodermal proliferation, we observed no such outcome when driving *UAS-twi-2x* with the *twi-GAL4* driver [40]. Instead, these embryos primarily appeared like wild-type, even though Twi overexpression did lead to an increase in Sna expression (Figure S6A,B). The only difference observable between control and Twi overexpression embryos was a slight asynchrony in the proliferation of mesodermal cells (compare Figure S6C–D″ to Figure S6E–F″). Based on mesodermal cell number and the condensed appearance of the chromatin, all control mesodermal cells were at either prophase or prometaphase (Figure S6C–D″). In contrast, PHH3 staining in embryos that overexpressed Twi exhibited subsets of mesodermal cells in anaphase or interphase, while other cells appeared to be in prophase or prometaphase (Figure S6E–F″). These results indicated that an increase of Twi activity levels disrupts mitotic synchrony and suggested that increased Twi activity may actually speed up the mitotic process in a subset of mesodermal cells.

### The Expression Of Snail In *twi^ry50^/twi^1^* Embryos Rescues the *twi* Mutant Phenotype

To ascertain whether the defective gastrulation and mesoderm phenotypes observed in *twi* hypomorphic mutants could be rescued by expression of Twi target genes, we used the GAL4-UAS system to overexpress Dmef2, Htl, and Sna in the embryo. We first examined the interplay between Twi and Sna, the only zygotically expressed factors that are essential for gastrulation. Previous work had shown that expression of Sna in a *twi* null background was able to partially rescue the mutant phenotype by inducing greater cell shape changes than a *twi* null background alone; however, Sna expression was unable to rescue gastrulation [35]. We confirmed these results by overexpressing Sna using *twi-Gal4* in *twi^1^* homozygous null embryos (Figure S7; data not shown). Similarly, overexpression of Htl or Dmef2 in *twi^1^* embryos did not lead to full rescue (Figure S8A,C). Embryos rescued with Dmef2, however, did exhibit extremely limited and disorganized muscle formation (Figure S8C). These experiments confirmed that Twi has several functions in gastrulation and showed that expression of these target genes separately in a *twi* null background was not sufficient to drive all the cellular functions needed to complete gastrulation.

**Figure 6:**
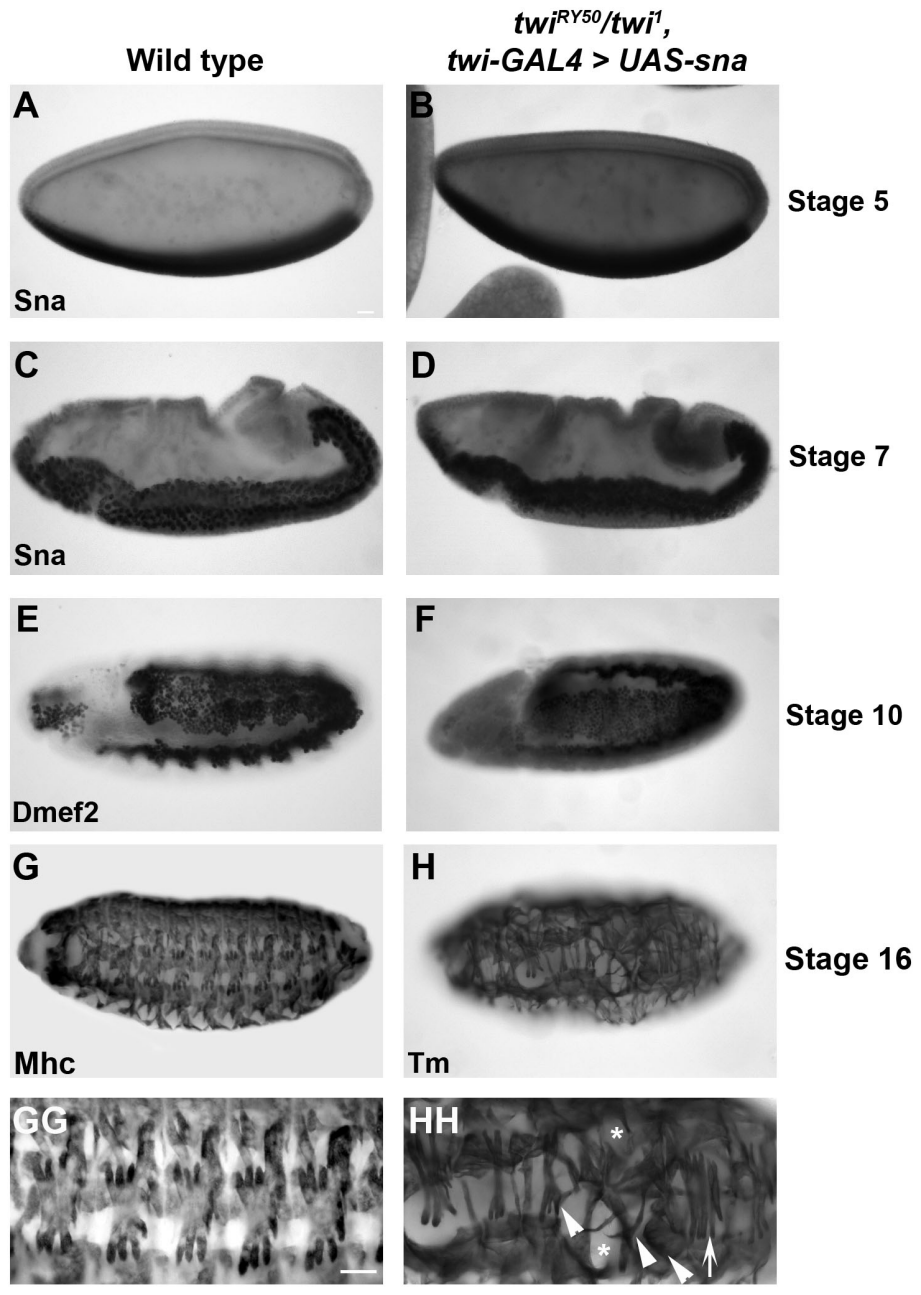
Sna overexpression can rescue gastrulation, mesodermal development and myogenesis in *twi^RY50^/twi^1^* embryos. Lateral views of *wild-type* (A,C,E,G,GG), and *twi^RY50^/twi^1^*, *twi-GAL4>UAS-sna* rescue (B,D,F,H,HH) embryos stained for Sna at stage 5 (A,B) and stage 7 (C,D), Dmef2 at stage 10 (E,F), Mhc (G-GG) and Tm at stage 16 (H-HH). Corresponding close-up images of embryos stained for Mhc or Tm are shown in GG and HH to show the final muscle pattern. Arrowheads indicate aberrant muscle morphologies, asterisks indicate muscle losses and arrows indicate duplicated lateral muscles (HH). Scale bars, 20 µm.

Based on these findings, we tested whether the expression of these target genes in a *twi* hypomorphic background could direct the changes necessary for gastrulation. We began by overexpressing Sna using the *twi-GAL4* driver in a *twi^RY50^/twi^1^* hypomorphic background and found, surprisingly, that these embryos underwent gastrulation and produced viable adult flies (Table S3). Normally, the *twi^RY50^/twi^1^* hypomorphic background was embryonic lethal, and no viable adults of this genotype were produced. Further analysis of the Sna-overexpressing *twi^RY50^/twi^1^* embryos revealed that gastrulation was fully rescued. Sna expression appeared wild-type (Figure 6A–D), despite the lack of mesoderm development that we observed in *twi^RY50^/twi^1^* embryos without Sna overexpression (compare Figure 6B,E with Figure 2H,K). Additionally, the invaginating cells expressed Twi, similar to wild-type embryos (Figure S7A–B). To better examine mesoderm formation in Sna-rescued *twi^RY50^/twi^1^* embryos, Dmef2 staining was performed. These embryos expressed a slightly reduced level of Dmef2, but the mesoderm spread and differentiated similar to wild-type embryos (Figure 6E–F, compare to Figure 3K). Analysis of the final muscle pattern revealed, strikingly, that Sna-rescued embryos formed most somatic muscles. We did detect slight disruptions in muscle patterning, including muscle loss as well as duplications of the lateral transverse (LT) muscles (Figures 68G–HH, S7C–EE, compare to Figure 1E). The LT muscles are particularly sensitive to the level of Twi activity, with duplications occurring when Twi levels are manipulated [40].

**Figure 7:**
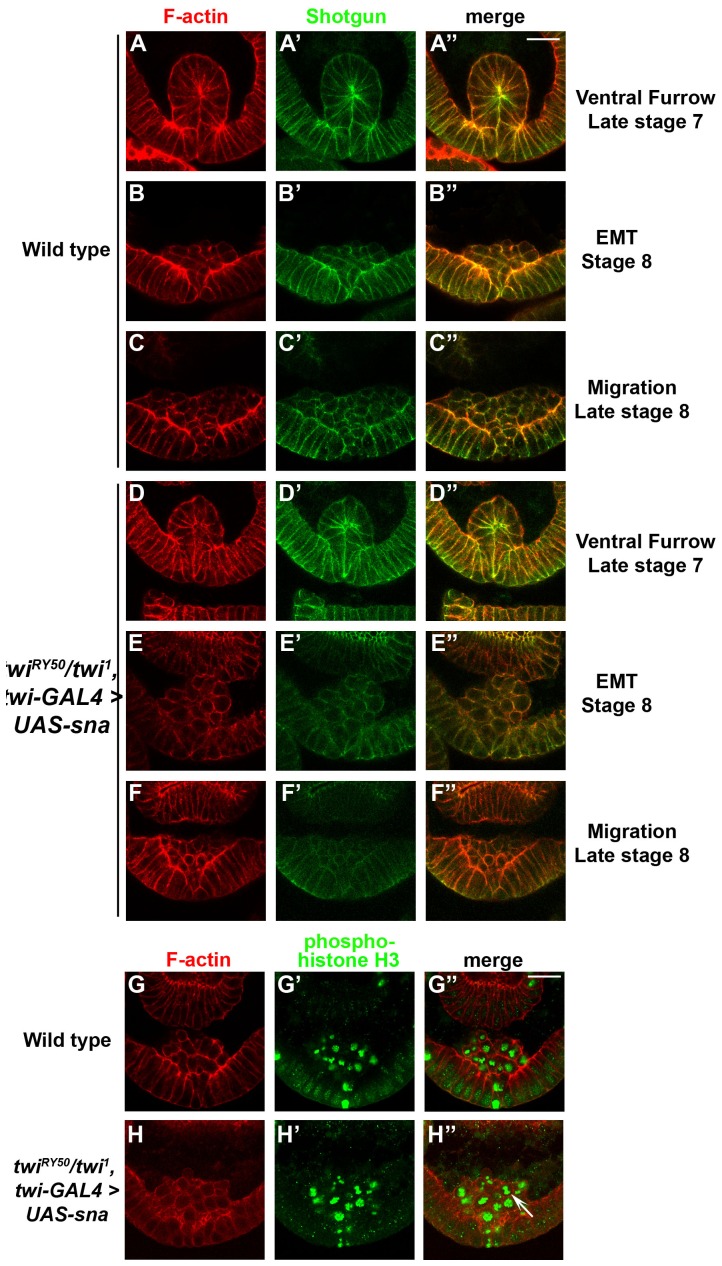
Sna overexpression in *twi^RY50^/twi^1^* embryos causes differences in VF size and mitotic synchrony. Transverse sections of wild-type (A–C″), control (G–G″) and *twi^RY50^/twi^1^*, *twi-GAL4>UAS-sna* rescue (D–F″, H–H″) embryos stained with phalloidin (red; A, B, C, D, E, F, H) and anti-Shotgun antibody (green; A′, B′, C′, D′, E′, F′) or anti PHH3 antibody (green; G′, H′). Colocalization of F-actin and shotgun appears yellow in merged panels (A″, B″, C″, D″, E″, F″). Merged panels of phalloidin and PHH3 are shown in G″ and H″. Late stage 7 (A–A″, D–D″), stage 8 (B–B″, E–E″, G–H″) and late stage 8 (C–C″, F–F″) embryos are shown. The white arrow indicates a mesodermal cell undergoing anaphase (H″). Scale bars 20 µm.

Transverse sections were used to determine whether the Sna-rescued *twi^RY50^/twi^1^* embryos exhibited developmental delays or reductions in VF size and mesodermal cell number. Curiously, despite the *twi^RY50^/twi^1^* background, Sna-rescued embryos did not exhibit a developmental delay and developed at approximately the same rate as wild-type embryos (Figure 7A–F″). Rescued embryos also displayed VFs that appeared wild-type, although the number of cells that make up the VF ranged from 12–15 cells, at an average of 13.8 cells, which was comparable to *twi^V50^* homozygous embryos (Figure 7D–D″; Table S2). The EMT and mesodermal migration also appeared like wild-type (Figure 7E–F″), with a slight reduction in DE-cadherin expression in mesodermal cells. Using PHH3, the Sna-rescued embryos also exhibited slightly asynchronous mesodermal mitosis (Figure 7H–H″). Together, we found that Sna overexpression in *twi^RY50^/twi^1^* embryos rescued gastrulation and mesodermal development, with the exception of slightly reduced VF sizes, asynchronous mitoses and minimal disruptions in the final muscle pattern. Overall, these results indicated that low Twi activity levels can be compensated by Sna overexpression, and that these low levels of activity were able to regulate processes essential for gastrulation and mesoderm formation.

We next examined the phenotypes of *twi^RY50^/twi^1^* embryos expressing *twi-GAL4>UAS-htl* or *twi-GAL4>UAS-Dmef2*. Overexpression of Htl rescued hindgut development (Figure S8B), and overexpression of Dmef2 resulted in limited rescue of the somatic muscles (Figure S8D). These embryos did not survive to adulthood (data not shown). These data suggested that Htl and Dmef2 can rescue certain aspects of mesoderm development in a *twi^RY50^/twi^1^* background, but not enough to restore viability, highlighting Sna's unique activity in the mesoderm.

## Discussion

Hypomorphic *twi* alleles were some of the earliest identified *Drosophila* embryonic mutants. Our thorough genetic characterization of these alleles and the establishment of a *twi* allelic series has helped to fine-tune our understanding of Twi function during gastrulation and mesodermal development. By genetically titrating Twi, we have gained insight to the mechanisms by which activation of Twi target genes translates to cellular process, such as mesoderm invagination, EMT, proliferation and migration. Finally, we show that expression of Twi target genes in the *twi^RY50^*/*twi^1^* background can rescue certain aspects of mesoderm and somatic muscle development. In the case of Sna, this rescue was nearly complete and included adult viability. These findings have deepened our understanding of how Twi controls multiple target genes during mesoderm and muscle development, and can be more broadly applied to vertebrate development and human cancer progression.

Based on embryonic phenotypes, the allelic series from least to most severe is: *twi^V50^/twi^V50^, twi^V50^/twi^1^, twi^RY50^/twi^RY50^, twi^RY50^/twi^1^*and *twi^1^/twi^1^.* Analysis of mutant embryos has shown that certain cellular processes have a greater sensitivity to the *twi* genetic background than others. We have previously shown that somatic myogenesis is exceptionally sensitive to Twi levels [40]. Similarly, the number of invaginated cells during gastrulation has a direct correspondence to the *twi* allelic series, with each step down yielding 2 fewer invaginated cells (Table S2). In contrast, formation of the ventral furrow is a robust process, with no disruption except for the strong allelic combinations of *twi^RY50^/twi^1^* and *twi^1^/twi^1^*. One way to explain our data is to assign different levels of Twi activity to these allelic combinations, with *twi^V50^/twi^V50^* having the greatest Twi activity level and *twi^RY50^/twi^1^* the least (and *twi^1^/twi^1^* having none). Following this logic, ventral furrow formation would require the least amount of *twi* activity and somatic myogenesis would require a large amount of activity. This explanation is complicated by the pleiotropic nature of Twi function, and the possibility that Twi acts in different ways at its gene targets to regulate their function in different cell types. Factors contributing to the differential response of particular Twi target genes could include: the number of Twi binding sites, the chromatin landscape, the Twi binding partner and the requirement for other cofactors. Further experiments will be required to assign specific cellular functions to particular *twi* alleles, and help us to elucidate the particular roles Twi plays in each discrete process.

An extension of this analysis provides a hypothesis for the developmental delays that occur in even the weakest Twi allelic combinations: inefficient activation of Twi target genes due to reduced Twi activity requires the embryo to put cell processes on hold until these gene products have built up sufficient expression to advance the process in question. This hypothesis is best illustrated in *twi^V50^*/*twi^1^* embryos, where levels of Htl and Dmef2 appear low (Figure 3C,F), fewer cells make up the VF to become mesodermal cells (Figures 4C–C′; S3C–C″, S4C–C″), and the development of these embryos is delayed (Table S1). Nevertheless, the final muscle pattern in *twi^V50^*/*twi^1^* embryos was relatively normal, with only some missing and mispatterned muscles (Figure 1C). This recovery suggests that, ultimately, mesoderm and muscle development is robust and can proceed in *twi* hypomorphs with a reduced number of founding mesodermal cells.

Though processes such as VF formation and muscle development were only affected in *twi* hypomorphs, synchronized mesoderm mitosis is one process that is disrupted in both *twi* hypermorphs and hypomorphs. Mesodermal mitosis occurs asynchronously in *twi^RY50^/twi^RY50^* embryos (Figure 5), as well as Twi overexpression embryos (Figure S6E–F″). Additionally, Sna rescue of *twi^RY50^/twi^1^* embryos (Figure 7G–H″) causes mesodermal mitosis to occur asynchronously. This sensitivity may be due to the Twi's role in both negatively and positively regulating the activity and expression of the cell cycle regulator String/cdc25 [34]. Previous work has shown that String, a Ser/Thr phosphatase, is precisely regulated by Twi to achieve synchronized cell divisions in the mesoderm [34], [46]. This finding has important implications for disease, as Twi has been shown to regulate cell proliferation both in cancer cells and mesenchymal stem cells [47], [48].

Our Sna rescue data also illustrates how Twi can function with other transcriptional regulators. Twi and Daughterless are examples of regulators that switch between transcriptional activation and repression depending on binding partner and tissue context [39], [40]. Moreover, Twi and Sna have been shown to concomitantly bind enhancers associated with *htl* and *tinman* [25], [36]. Independently, Sna has been shown to repress number of genes, such as *single-minded*, *rhomboid*, *wntD* and *short gastrulation*, in the ventral-most mesodermal cells, restricting their expression to lateral regions [7], [29], [49]. The repression of these target genes, however, functions to promote gastrulation, as Sna is essential for the initiation of cell ^1^shape changes, even in the absence of Twi [35]. For example, *twi* null mutant embryos, which briefly express Sna in a Dl-dependent/Twi-independent manner, exhibit cell shape changes that are required for mesodermal invagination. These cell shape changes, however, are entirely missing in *twi/sna* double mutants [35].

**Figure 8:**
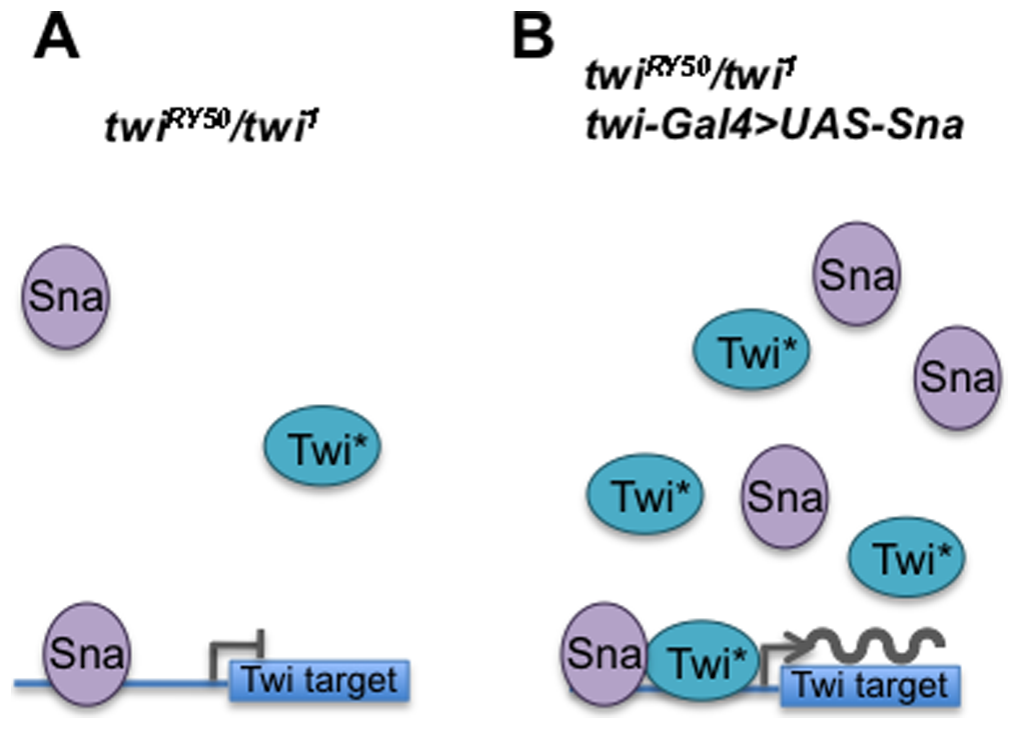
A model for Sna overexpression rescue of *twi^RY50^/twi^1^* hypomorphs. (A) In *twi^RY50^/twi^1^* embryos without Sna rescue (left), expression levels of Twi^RY50^ (Twi*) and Sna are low, reducing the likelihood that these proteins will interact or bind to activate their target genes. These circumstances lead to no mesoderm formation or viability. (B) In *twi^RY50^/twi^1^* embryos where Sna is overexpressed (right), an increased amount of Twi^RY50^ protein is available to cooperate with the increased levels of Sna to activate their gene targets. Thereby, Sna overexpression leads to mesoderm formation and viability.

Full Sna rescue was specific to the *twi^RY50^/twi^1^* hypomorphic background, while Sna overexpression in *twi^1^* homozygous null embryos had a limited rescue function [35]. What mechanism could explain the ability of Sna overexpression to rescue hypomorphic mutant embryos, but not homozygous null embryos? While Sna was initially characterized as a transcriptional repressor, recent work has uncovered a role for Sna in the activation of mesodermal target genes [25]. A subset of Sna target genes, including *Dmef2, htl and tin*, are positively regulated by both Twi and Sna [14], [25]. We know, however, from the inability of overexpressed Sna to rescue gastrulation in *twi^1^* homozygous mutants that this Sna activation of target genes is not sufficient [35] Additionally, Sna binds to the Twi enhancer and positively regulates its transcription [25]. Consistent with this finding, we observed that *twi^RY50^/twi^1^* embryos rescued by Sna overexpression showed wild-type levels of Twi expression (Figure S7 and data not shown). These results suggested one possible model where Sna rescue increases *twi^RY50^* expression levels, thereby providing sufficient Twi activity to drive gastrulation and mesoderm formation in combination with Sna (Figure 8). Given the data from recent whole genome studies, this rescue is likely direct [25].

Gastrulation relies on the level of Twi activity to regulate the EMT and cell migration. Strikingly, in *twi^RY50^*/*twi^1^* embryos, invaginated mesodermal cells appeared to undergo EMT initially, but, at later stages, these mesodermal cells were no longer observed (Figures 4E′–E″, S4E–E″; S5E–E″). Because apoptosis was not detected in these embryos, one possible explanation is that the *twi^RY50^*/*twi^1^* mesodermal cells underwent a mesenchymal-to-epithelial transition (MET) to revert back to their epithelial morphology. This effect suggests one role of Twi is to prevent the EMT from reverting. This finding has important implications for human cancers, suggesting that therapeutic knockdown of Twi could be crucial for halting the initiation of metastasis. The MET observed in *twi^RY50^*/*twi^1^* embryos is also relevant to the next step of metastasis, as recent studies have found that metastatic cancer cells must revert back to an epithelial state in order to proliferate and form secondary tumors (reviewed in [50]). Another possibility is that *twi^RY50^*/*twi^1^* mesodermal cells are unable to maintain mesodermal cell fate and no longer express mesodermal markers. This possibility suggests that the cells have dedifferentiated, a process that is relevant for tumor formation and the development of cancer stem cells (reviewed in [61]). Overall, these results highlight the parallels between mesodermal development and tumor formation, which suggests that nuanced regulation of Twi is also critical for different stages of tumor development and metastasis. In fact, Twi and its target genes, particularly Sna, have been implicated in various metastatic tumors including breast, esophageal and uterine cancers [50], [51]. Additionally, expression of Twi in human tumors correlates to resistance to a number of chemotherapeutics as well as poor outcomes [52]–[54]. Similar to their role in *Drosophila* gastrulation, both the Twist and Snail families of proteins control genes that direct cell shape changes and EMT in humans [50], [51].

Finally, the allelic series developed in this study has provided a tractable genetic system for the study of other factors affecting cell shape changes, the EMT, cell proliferation and cell cycle regulation. Our results are quantitative and provide benchmarks such as the number of invaginated cells in each genetic background. Therefore, this system provides an extremely sensitive *in vivo* read-out for testing the role of other genes, and potentially drugs, that affect processes directly relevant to human cancers.

## Materials and Methods

### Drosophila Genetics

The following fly stocks were used: *twi^1^/SM6 Cy Roi eve-LacZ* [6], *twi^V50^/SM6 Cy Roi eve-LacZ* [9], *twi^RY50^/SM6 Cy Roi eve-LacZ* [9], *twi-GAL4* [55], *UAS-twi* [56], *UAS-twi 2x* [56], *UAS-sna* (gift of Y. Nibu; [57]), *UAS-Htl* [58] and *UAS-Dmef2* [59]. The GAL4-UAS system [45] was used for expression studies. All genetic crosses were performed at 25°C. *yw* or *OreR* were used as wild-type strains in all immunohistochemistry images except in transverse section images stained for phospho-Histone H3 (PHH3), where *twi^RY50^/+* embryos represented control images. Embryos were staged according to Campos-Ortega and Hartenstein (1985). Approximate ages are: stage 5, 2:10–2:50 h after egg laying (AEL); stage 7, 3:00–3:10 h AEL; stage 8, 3:10–3:40 h AEL; stage 9, 3:40–4:20 h AEL; stage 10, 4:20–5:20 AEL; and stage 16, 13:00–16:00 AEL. For hypomorphic allelic combinations that experience developmental delays, embryos were compared in which the ventral furrow (VF) was at its maximum.

### Immunohistochemistry And Imaging

Whole mount immunohistochemistry was performed following standard techniques [60]. Certain antibodies were preabsorbed (PA) against fixed *yw* embryos and used in combination with the Tyramide Signal Amplification system (TSA; PerkinElmer Life Sciences). The following antibodies were used: anti-Mhc (1∶10,000; TSA; D. Kiehart), anti-tropomyosin (Tm) (1∶1000, AbCam), anti-Sna (1∶200; Y. Nibu), anti-Htl (1∶2000; TSA; A. Michelson), anti-Dmef2 (1∶200; B. Patterson), anti-β-galactosidase (β-gal) (1∶1000; Promega). Biotinylated secondary antibodies (1∶200) were used in combination with the Vector Elite ABC kit (Vector Laboratories, CA) and diaminobenzidine (DAB) stain. Specimens were embedded in Araldite. Images were captured using an Axiocam digital camera (Zeiss). All DAB mesodermal images are a merge of several focal planes and were combined into one image using Adobe Photoshop software.

Fluorescent immunohistochemistry was conducted using extended one hour fixes in a solution of 4% formaldehyde in sodium phosphate buffer. Embryos were then treated with the following primary antibodies: anti-Shotgun (1∶100; Developmental Studies Hybridoma Bank, Univ. of Iowa), anti-β-gal (1∶1000; Promega) and anti-PHH3 (1∶100, Upstate Biotechnology). Primary antibodies were detected using Alexa 488-, Alexa 555- or Alexa 647-conjugated secondary antibodies (1∶400; Invitrogen). Alexa 555-conjugated phalloidin was used to visualize F-actin (1∶200; Invitrogen). Embryos were mounted in Vectashield (Vector Laboratories, CA) or, for phalloidin stainings, ProLong Gold antifade reagent (Invitrogen).

Double or triple staining using anti-β-gal (1∶1000; Promega) was used for both immunohistochemistry and fluorescent immunohistochemistry to identify the presence of lacZ-marked balancer chromosomes.

Transverse sections of embryos were dissected with number 15 scalpels and mounted in ProLong Gold. Images were acquired at least 10 µm from the edge of the tissue section to avoid capturing images of damaged tissue. In all of these experiments, staging was based on developmental processes that occur independently of mesodermal development, such as cephalic furrow formation, pole cell migration, dorsal fold formation and midgut invagination [27], [35]. Confocal micrographs were acquired on a Zeiss LSM510 confocal scanning system mounted on an Axiovert 100 M microscope with a 63x 1.2NA C-Apochromat water objective. Images were processed using Zeiss LSM Image Browser software.

## Supporting Information

Figure S1 ***twi^RY50^/twi^1^*** **embryos exhibit a reduced number of Sna-expressing cells.** Ventral views of a wild-type (A) and a *twi^RY50^/twi^1^* (B) embryo are shown. Both embryos have been stained with anti-Sna antibody. Scale bar, 20 µm.
(TIF)

Figure S2 **A reduction in Twi activity causes a developmental delay.** Confocal micrographs of transverse sections of embryos are shown. Wild-type (A,C,E) and *twi^1^/twi^1^* (B,D,F) embryos at late stage 7 (A,B), stage 8 (C,D), and late stage 8 (E,F) are shown. Embryos have been stained for phalloidin to visualize F-actin. Scale bar, 20 µm.
(TIF)

Figure S3 **Ventral furrow formation directly responds to Twi activity levels.** Confocal micrographs of transverse sections of *wild-type* (A–A″), *twi^V50^/twi^V50^* (B–B″), *twi^V50^/twi^1^* (C–C″), *twi^RY50^/twi^RY50^* (D–D″), *twi^RY50^/twi^1^* (E–E″), and *twi^1^/twi^1^* (F–F″) embryos stained with phalloidin to visualize F-actin (red; A, B, C, D, E, F) and an antibody raised against Shotgun (green; A′, B′, C′, D′, E′, F′). Merged panels are shown in A″, B″, C″, D″, E″, F″. Phalloidin and Shotgun expression reveal cell shapes. Shotgun expression is enriched at sites of apical constriction. All embryos are at stage 8, except the wild-type embryo, which is at late stage 7. Scale bar, 20 µm.
(TIF)

Figure S4 **The epithelial to mesenchymal transition (EMT) relies on Twi.** Transverse sections of *wild-type* (A–A″), *twi^V50^/twi^V50^* (B–B″), *twi^V50^/twi^1^* (C–C″), *twi^RY50^/twi^RY50^* (D–D″), *twi^RY50^/twi^1^* (E–E″), and *twi^1^/twi^1^* (F–F″) embryos stained with phalloidin (red; A, B, C, D, E, F) and anti-Shotgun antibody (green; A′, B′, C′, D′, E′, F′). Merged panels (A″, B″, C″, D″, E″, F″) are shown, where colocalization of F-actin and Shotgun appears yellow. Shotgun expression is higher in the ectodermal cells (white arrowheads) than in the mesodermal cells undergoing the EMT (white arrows; panels A′, B′, C′, D′, E′, and F′). Mesodermal cells that have contacted the ectodermal cells are indicated by concave arrowheads in panels A″, B″, C″, D″, and E″. The wild-type embryo is at stage 8, the *twi^V50^/twi^V50^* embryo is at early stage 9, and all other embryos are at stage 9. Scale bar, 20 µm.
(TIF)

Figure S5 **Cell division and mesodermal migration are sensitive to Twi.** Transverse sections of *wild-type* (A–A″), *twi^V50^/twi^V50^* (B–B″), *twi^V50^/twi^1^* (C–C″), *twi^RY50^/twi^RY50^* (D–D″), *twi^RY50^/twi^1^* (E–E″), and *twi^1^/twi^1^* (F–F″) embryos stained with phalloidin (red; A, B, C, D, E, F) and anti-Shotgun antibody (green; A′, B′, C′, D′, E′, F′). Merged panels (A″, B″, C″, D″, E″, F″) are shown, where colocalization of F-actin and Shotgun appears yellow. Shotgun expression is higher in the ectodermal cells (white arrowheads) than in the mesodermal cells undergoing the EMT (white arrows; panels A′, B′, C′, D′, E′, and F′). Mesodermal cells that have contacted the ectodermal cells are indicated by concave arrowheads in panels A″, B″, C″, D″, and E″. The wild-type embryo is at stage 8, the *twi^V50^/twi^V50^* embryo is at early stage 9, and all other embryos are at stage 9. Scale bar, 20 µm.
(TIF)

Figure S6 **Overexpression of Twi in a wild-type background causes increases in Sna expression and asynchrony in mitotic mesodermal cells.** Lateral views (A–B) and transverse sections (C–F″) of wild-type (A), control (C–D″) and *twi-GAL4 > UAS-twi 2X* (B, E–F″) embryos are shown. Stage 7 embryos were stained for Sna and Eve to show Sna expression levels and to precisely stage embryos, respectively (A, B). Transverse sections of stage 8 embryos were stained with phalloidin (red; C, D, E, F) and anti-PHH3 (green; C′, D′, E′, F′) antibodies. Merged panels are shown in C″, D″, E″, F″. White arrows indicate cells in anaphase and white arrowheads indicate cells that do not express PHH3. Scale bars, 20 µm.
(TIF)

Figure S7 **Sna overexpression in** ***twi^RY50^/twi^1^*** **embryos results in nearly wild-type muscle morphology and patterning.** Lateral views of wild-type (A, C, CC) and *twi^RY50^/twi^1^*, *twi-GAL4>UAS-sna* rescue (B, D, DD, E, EE) embryos stained for Twi expression at stage 8 (A,B), Mhc (C, CC) and Tm at stage 16 (D-EE). Arrowheads indicate aberrant muscle morphologies, asterisks indicate muscle losses and arrows indicate duplicated lateral transverse muscles. Scale bar, 20 µm.
(TIF)

Figure S8 **Overexpression of Htl and Dmef2 in** ***twi1*** **and** ***twi1/twiRY50*** **mutants can rescue limited aspects of mesoderm development.** Lateral views of *twi^1^/twi^1^*, *twi-GAL4>UAS-htl* (A) and *twi^1^/twi^1^*, *twi-GAL4>UAS-Dmef2* (B) rescue embryos are shown. Dorsal views of and *twi^RY50^/twi^1^*, *twi-GAL4>UAS-htl* (B) and and *twi^RY50^/twi^1^*, *twi-GAL4>UAS-Dmef2* rescue embryos are shown. All embryos have been stained for Tm to observe the final muscle pattern. An arrow indicates the hindgut structure (B) and arrowheads show TM positive muscles that have formed (C, D). Scale bar, 20 µm.
(TIF)

Table S1 **The effect of Twist activity levels on developmental time during gastrulation and mesodermal migration at 25°C.**
(DOCX)

Table S2 **The effect of** ***twist*** **hypomorphic alleles and Twist activity levels on the number of invaginated mesodermal cells during gastrulation.**
(DOCX)

Table S3 **Rescue of adult flies by** ***UAS-sna*** **overexpression in a** ***twi*** **mutant background.**
(DOCX)
