# Supplementary figures and images for: Discrete Levels of Twist Activity Are Required to Direct Distinct Cell Functions during Gastrulation and Somatic Myogenesis
Source: PLoS One. 2014 Jun 10;9(6):e99553. doi: 10.1371/journal.pone.0099553 (PMC4051702; doi:10.1371/journal.pone.0099553)

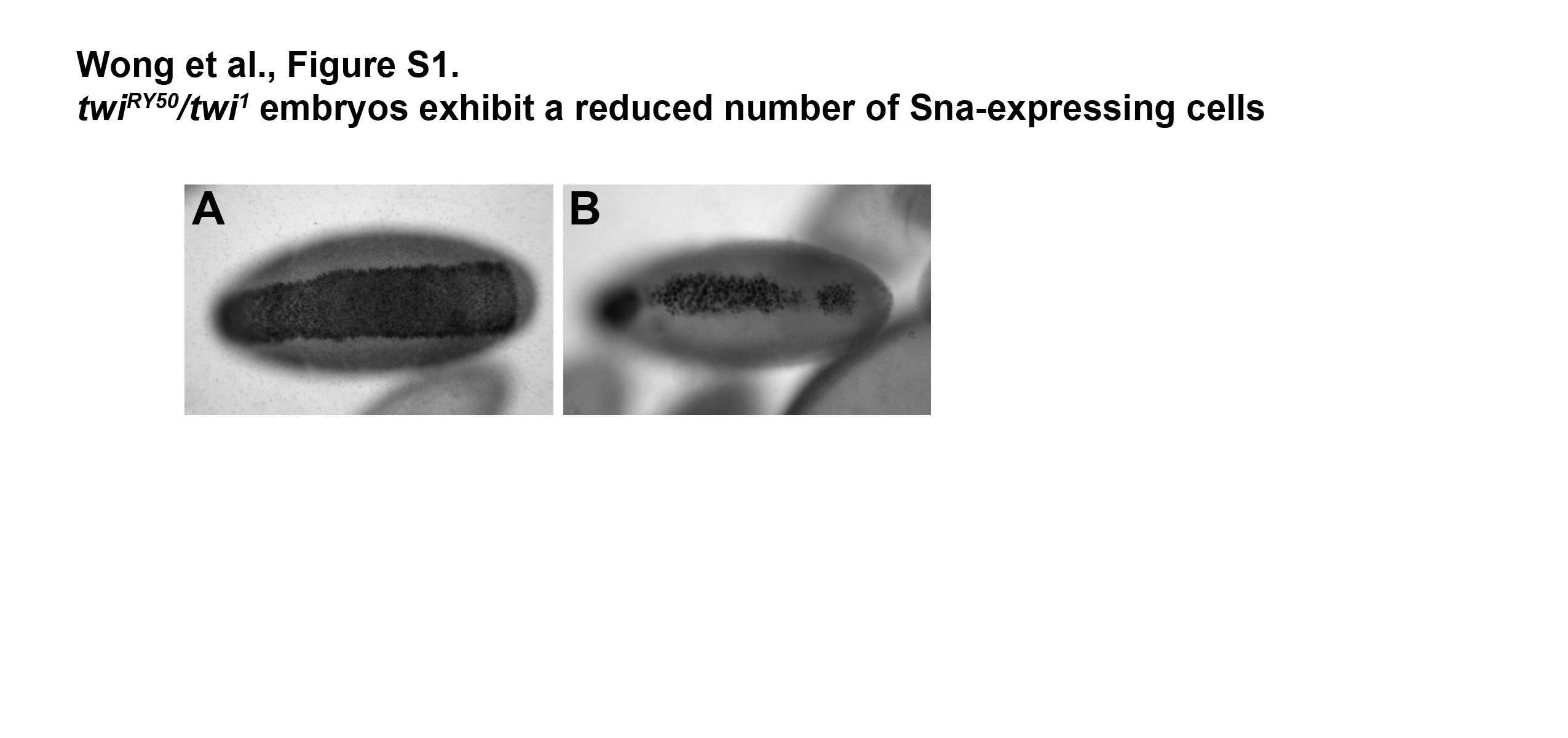

Supplement: Figure S1 — twiRY50/twi1 embryos exhibit a reduced number of Sna-expressing cells. Ventral views of a wild-type (A) and a twiRY50/twi1 (B) embryo are shown. Both embryos have been stained with anti-Sna antibody. Scale bar, 20 µm. (TIF) [file pone.0099553.s001.tif]

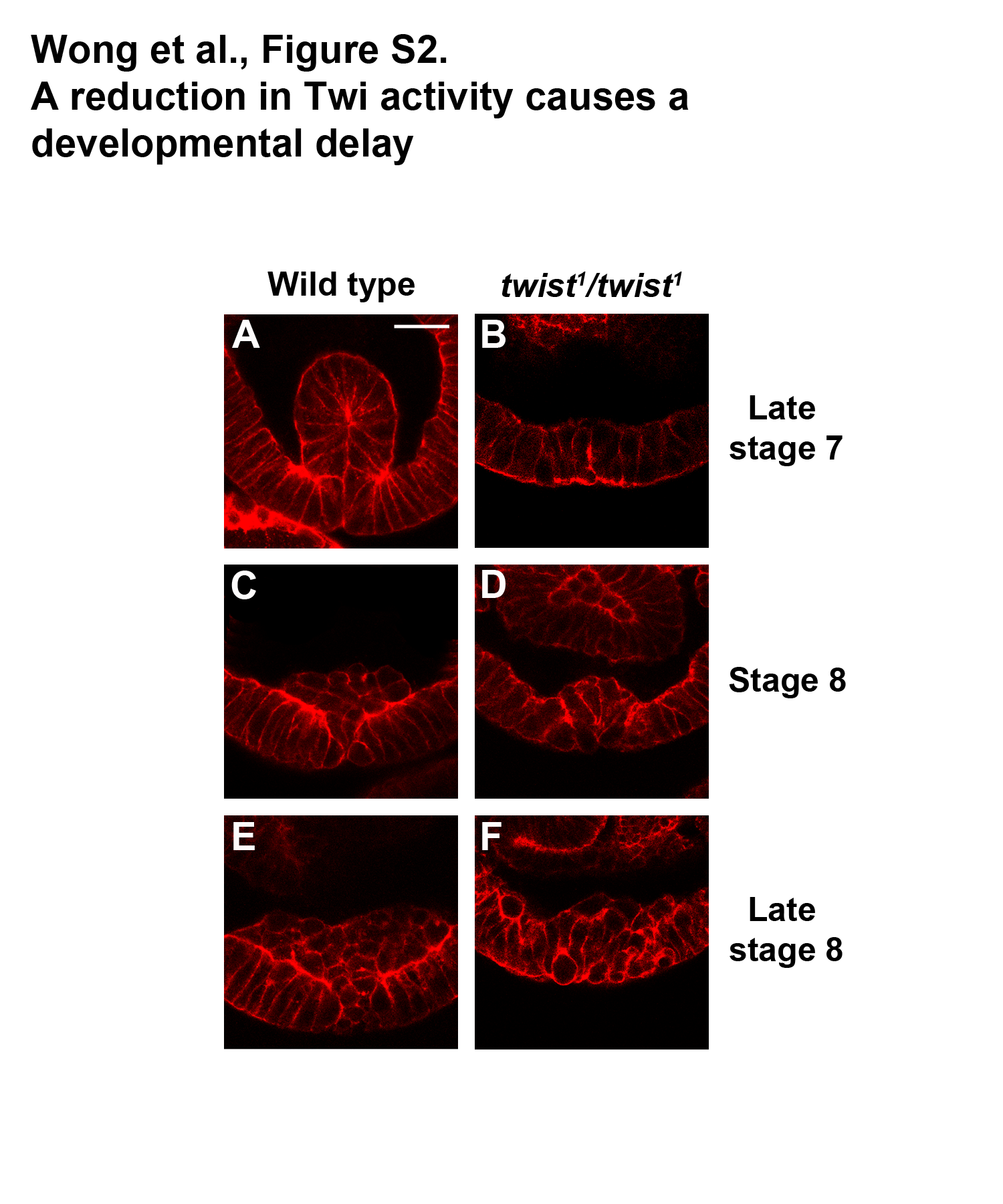

Supplement: Figure S2 — A reduction in Twi activity causes a developmental delay. Confocal micrographs of transverse sections of embryos are shown. Wild-type (A,C,E) and twi1/twi1 (B,D,F) embryos at late stage 7 (A,B), stage 8 (C,D), and late stage 8 (E,F) are shown. Embryos have been stained for phalloidin to visualize F-actin. Scale bar, 20 µm. (TIF) [file pone.0099553.s002.tif]

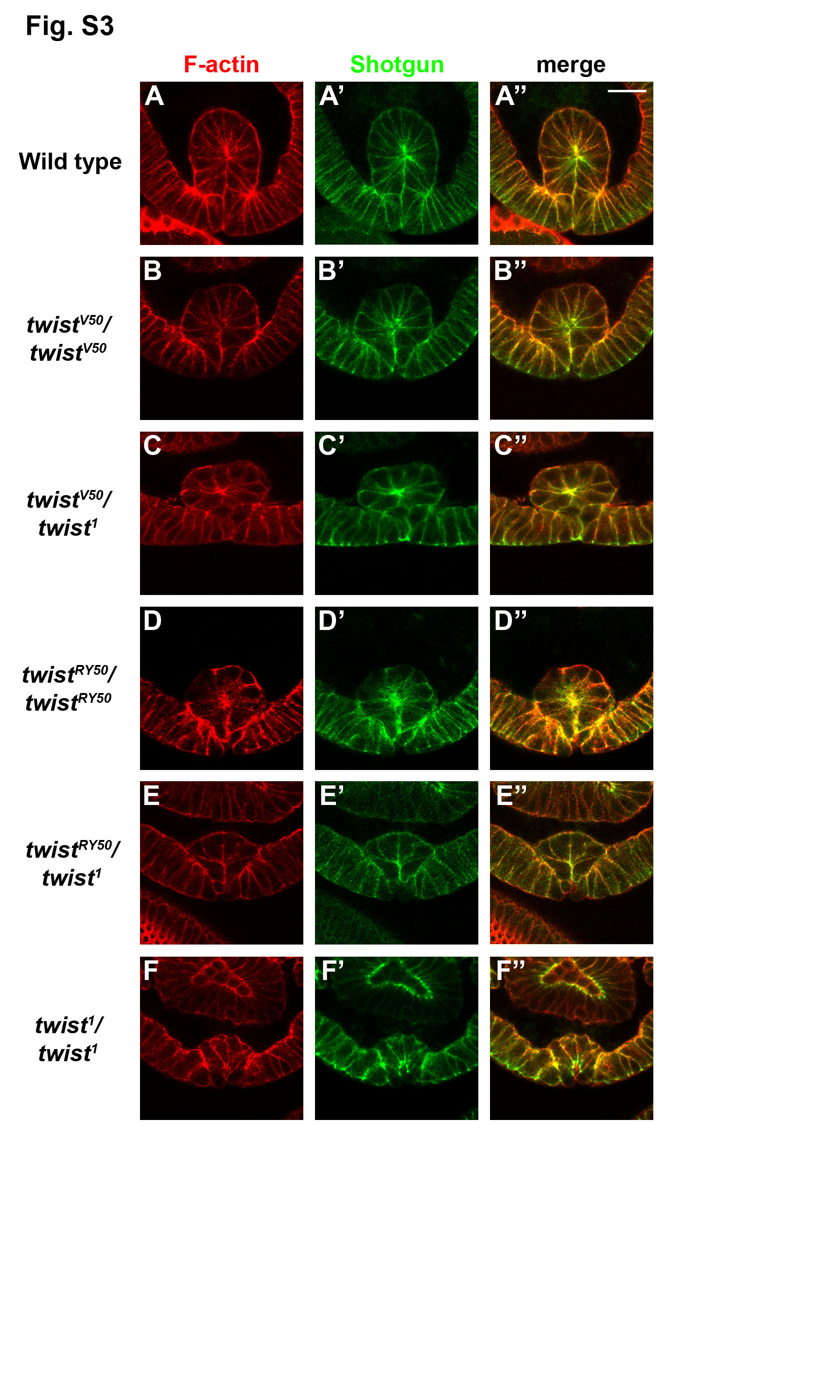

Supplement: Figure S3 — Ventral furrow formation directly responds to Twi activity levels. Confocal micrographs of transverse sections of wild-type (A–A″), twiV50/twiV50 (B–B″), twiV50/twi1 (C–C″), twiRY50/twiRY50 (D–D″), twiRY50/twi1 (E–E″), and twi1/twi1 (F–F″) embryos stained with phalloidin to visualize F-actin (red; A, B, C, D, E, F) and an antibody raised against Shotgun (green; A′, B′, C′, D′, E′, F′). Merged panels are shown in A″, B″, C″, D″, E″, F″. Phalloidin and Shotgun expression reveal cell shapes. Shotgun expression is enriched at sites of apical constriction. All embryos are at stage 8, except the wild-type embryo, which is at late stage 7. Scale bar, 20 µm. (TIF) [file pone.0099553.s003.tif]

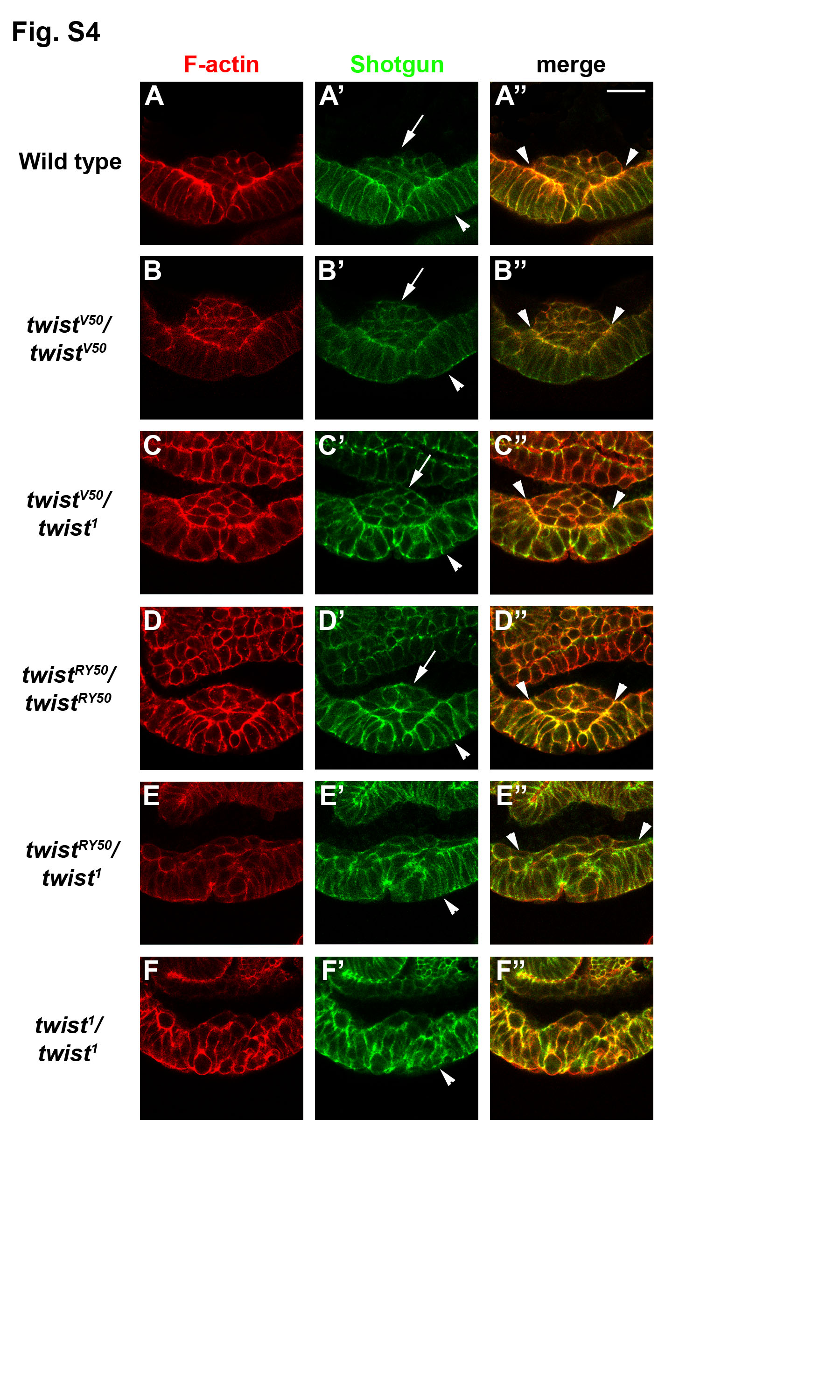

Supplement: Figure S4 — The epithelial to mesenchymal transition (EMT) relies on Twi. Transverse sections of wild-type (A–A″), twiV50/twiV50 (B–B″), twiV50/twi1 (C–C″), twiRY50/twiRY50 (D–D″), twiRY50/twi1 (E–E″), and twi1/twi1 (F–F″) embryos stained with phalloidin (red; A, B, C, D, E, F) and anti-Shotgun antibody (green; A′, B′, C′, D′, E′, F′). Merged panels (A″, B″, C″, D″, E″, F″) are shown, where colocalization of F-actin and Shotgun appears yellow. Shotgun expression is higher in the ectodermal cells (white arrowheads) than in the mesodermal cells undergoing the EMT (white arrows; panels A′, B′, C′, D′, E′, and F′). Mesodermal cells that have contacted the ectodermal cells are indicated by concave arrowheads in panels A″, B″, C″, D″, and E″. The wild-type embryo is at stage 8, the twiV50/twiV50 embryo is at early stage 9, and all other embryos are at stage 9. Scale bar, 20 µm. (TIF) [file pone.0099553.s004.tif]

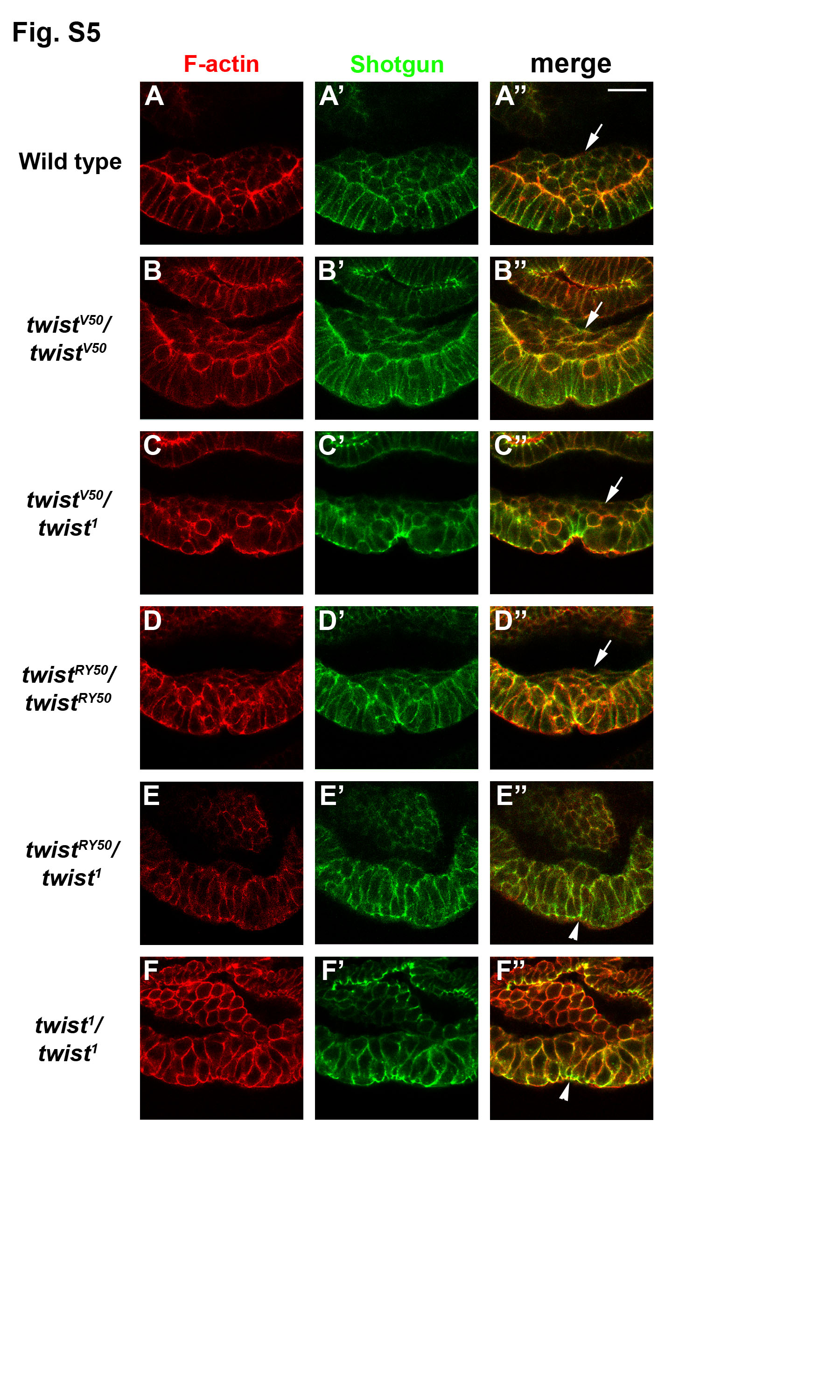

Supplement: Figure S5 — Cell division and mesodermal migration are sensitive to Twi. Transverse sections of wild-type (A–A″), twiV50/twiV50 (B–B″), twiV50/twi1 (C–C″), twiRY50/twiRY50 (D–D″), twiRY50/twi1 (E–E″), and twi1/twi1 (F–F″) embryos stained with phalloidin (red; A, B, C, D, E, F) and anti-Shotgun antibody (green; A′, B′, C′, D′, E′, F′). Merged panels (A″, B″, C″, D″, E″, F″) are shown, where colocalization of F-actin and Shotgun appears yellow. Shotgun expression is higher in the ectodermal cells (white arrowheads) than in the mesodermal cells undergoing the EMT (white arrows; panels A′, B′, C′, D′, E′, and F′). Mesodermal cells that have contacted the ectodermal cells are indicated by concave arrowheads in panels A″, B″, C″, D″, and E″. The wild-type embryo is at stage 8, the twiV50/twiV50 embryo is at early stage 9, and all other embryos are at stage 9. Scale bar, 20 µm. (TIF) [file pone.0099553.s005.tif]

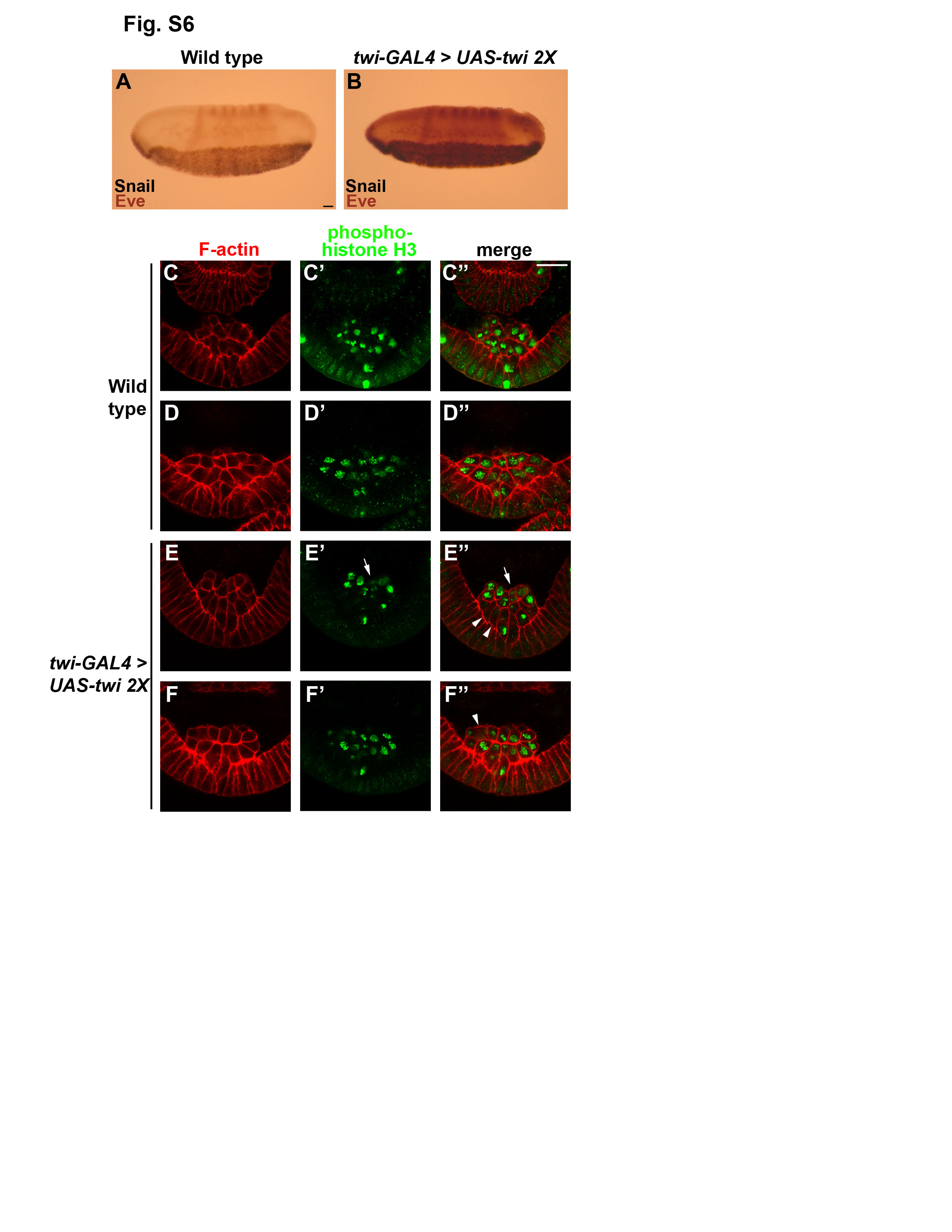

Supplement: Figure S6 — Overexpression of Twi in a wild-type background causes increases in Sna expression and asynchrony in mitotic mesodermal cells. Lateral views (A–B) and transverse sections (C–F″) of wild-type (A), control (C–D″) and twi-GAL4 > UAS-twi 2X (B, E–F″) embryos are shown. Stage 7 embryos were stained for Sna and Eve to show Sna expression levels and to precisely stage embryos, respectively (A, B). Transverse sections of stage 8 embryos were stained with phalloidin (red; C, D, E, F) and anti-PHH3 (green; C′, D′, E′, F′) antibodies. Merged panels are shown in C″, D″, E″, F″. White arrows indicate cells in anaphase and white arrowheads indicate cells that do not express PHH3. Scale bars, 20 µm. (TIF) [file pone.0099553.s006.tif]

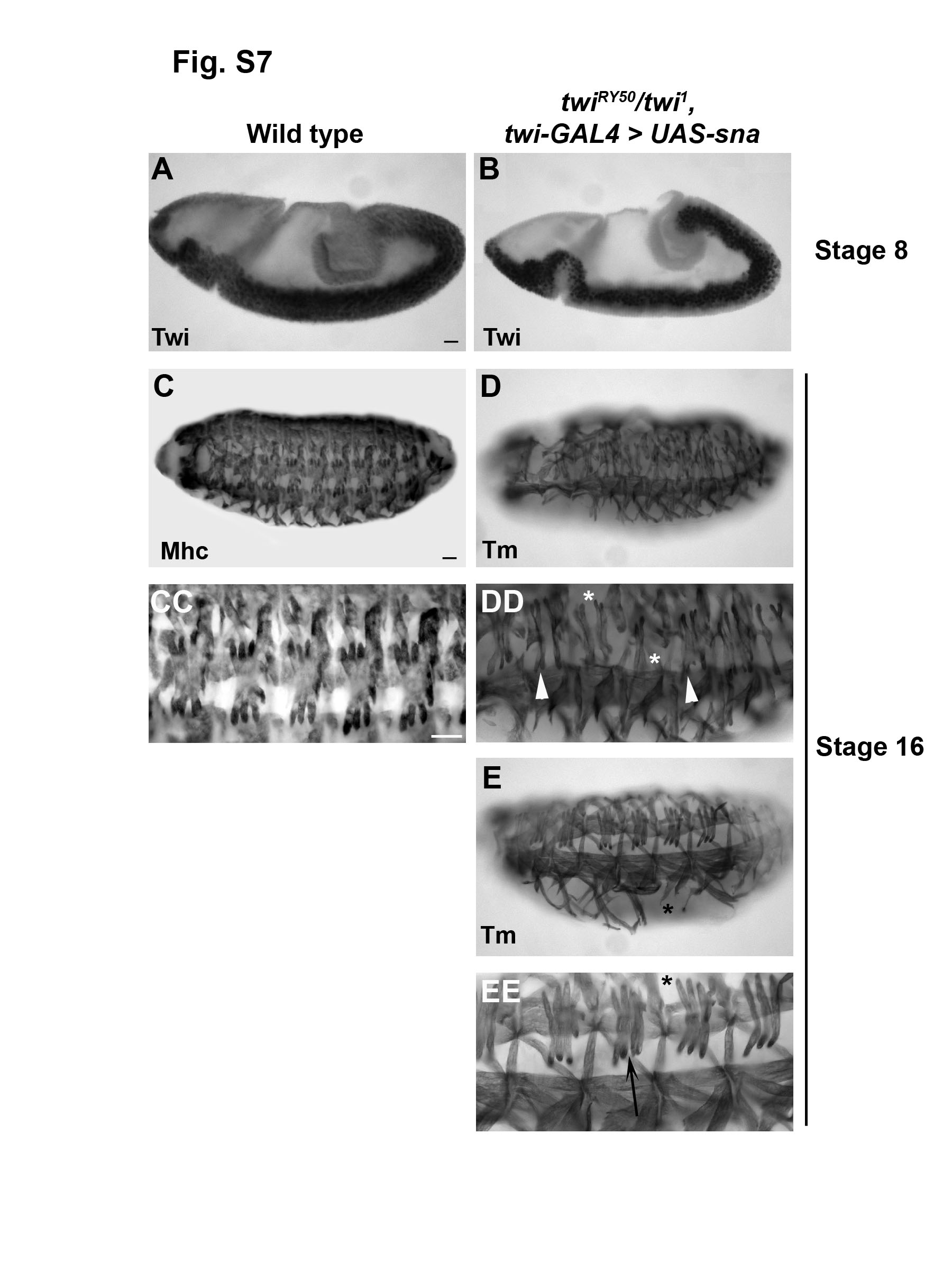

Supplement: Figure S7 — Sna overexpression in twiRY50/twi1 embryos results in nearly wild-type muscle morphology and patterning. Lateral views of wild-type (A, C, CC) and twiRY50/twi1, twi-GAL4>UAS-sna rescue (B, D, DD, E, EE) embryos stained for Twi expression at stage 8 (A,B), Mhc (C, CC) and Tm at stage 16 (D-EE). Arrowheads indicate aberrant muscle morphologies, asterisks indicate muscle losses and arrows indicate duplicated lateral transverse muscles. Scale bar, 20 µm. (TIF) [file pone.0099553.s007.tif]

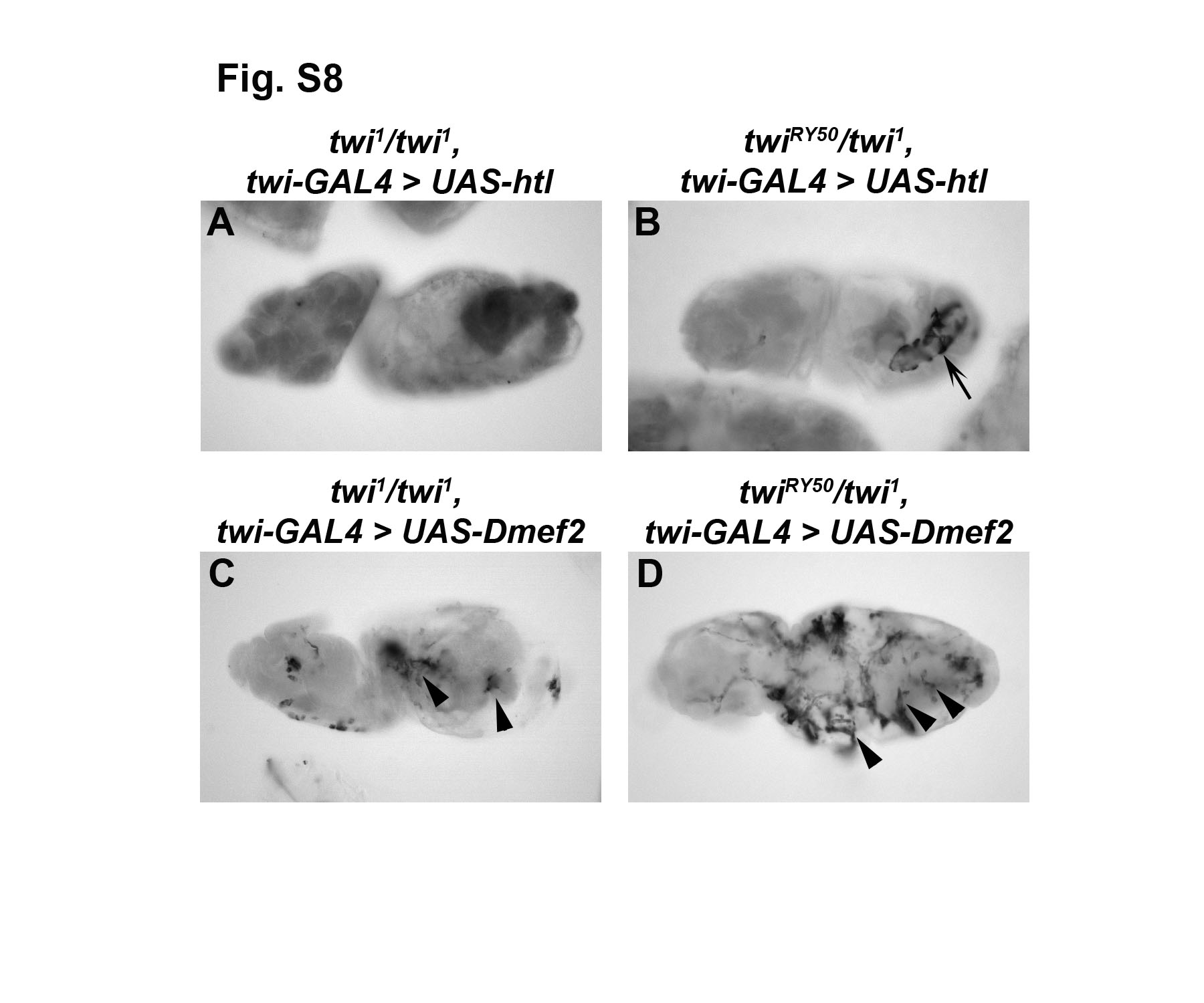

Supplement: Figure S8 — Overexpression of Htl and Dmef2 in twi1 and twi1/twiRY50 mutants can rescue limited aspects of mesoderm development. Lateral views of twi1/twi1, twi-GAL4>UAS-htl (A) and twi1/twi1, twi-GAL4>UAS-Dmef2 (B) rescue embryos are shown. Dorsal views of and twiRY50/twi1, twi-GAL4>UAS-htl (B) and and twiRY50/twi1, twi-GAL4>UAS-Dmef2 rescue embryos are shown. All embryos have been stained for Tm to observe the final muscle pattern. An arrow indicates the hindgut structure (B) and arrowheads show TM positive muscles that have formed (C, D). Scale bar, 20 µm. (TIF) [file pone.0099553.s008.tif]
