# Supplementary material for: Discrete Levels of Twist Activity Are Required to Direct Distinct Cell Functions during Gastrulation and Somatic Myogenesis
Source: PLoS One. 2014 Jun 10;9(6):e99553. doi: 10.1371/journal.pone.0099553 (PMC4051702; doi:10.1371/journal.pone.0099553)
Supplement: Table S1 — The effect of Twist activity levels on developmental time during gastrulation and mesodermal migration at 25°C. (DOCX) [file pone.0099553.s009.docx]

**Table S1. The effect of Twist activity levels on developmental time during gastrulation and mesodermal migration at 25˚C.**

|  | **VF formation** | | **EMT** | | **Cell proliferation & migration** | |
| --- | --- | --- | --- | --- | --- | --- |
| **Genotype** | **Stage completed** | **Approx. time (minutes)** | **Stage completed** | **Approx. time (minutes)** | **Stage completed** | **Approx. time (minutes)** |
| Wildtype | Late stage 7 | 20 | Stage 8 | 15 | Late stage 8 | 15 |
| *twist^V50^/twist^V50^* | Stage 8 | 35 | Early stage 9 | 20 | Stage 9 | 20 |
| *twist^V50^/twist^1^* | Stage 8 | 35 | Stage 9 | 30 | Late stage 9 | 35 |
| *twist^RY50^/twist^RY50^* | Stage 8 | 35 | Stage 9 | 30 | Late stage 9 | 35 |
| *twist^RY50^/twist^1^* | Stage 8 | 35 | Stage 9 | 30 | Late stage 9 | 35 |
| *twist^1^/twist^1^* | Stage 8 | 35 | Stage 9 | 30 | Late stage 9 | 35 |
