# Supplementary material for: Discrete Levels of Twist Activity Are Required to Direct Distinct Cell Functions during Gastrulation and Somatic Myogenesis
Source: PLoS One. 2014 Jun 10;9(6):e99553. doi: 10.1371/journal.pone.0099553 (PMC4051702; doi:10.1371/journal.pone.0099553)
Supplement: Table S2 — The effect of twist hypomorphic alleles and Twist activity levels on the number of invaginated mesodermal cells during gastrulation. (DOCX) [file pone.0099553.s010.docx]

**Table S2. The effect of *twist* hypomorphic alleles and Twist activity levels on the number of invaginated mesodermal cells during gastrulation.**

| **Genotype** | **n (number of embryos)** | **Observed range of invaginated cells** | **Average number of invaginated cells** |
| --- | --- | --- | --- |
| *twist-GAL4>UAS-twi 2X* | 3 | 15-16 | 15.7 |
| Wildtype | 5 | 15-18 | 16.4 |
| *twist^V50^/twist^V50^* | 6 | 12-16 | 13 |
| *twist^V50^/twist^1^* | 8 | 9-13 | 11 |
| *twist^RY50^/twist^RY50^* | 6 | 8-10 | 9.3 |
| *twist^RY50^/twist^1^* | 3 | 6-8 | 7.3 |
| *twist^1^/twist^1^* | 4 | 6-8 | 7.5 |
| *twi-GAL4>UAS-sna, twist^RY50^/twist^1^* | 4 | 12-14 | 13.8 |
