# Supplementary material for: Discrete Levels of Twist Activity Are Required to Direct Distinct Cell Functions during Gastrulation and Somatic Myogenesis
Source: PLoS One. 2014 Jun 10;9(6):e99553. doi: 10.1371/journal.pone.0099553 (PMC4051702; doi:10.1371/journal.pone.0099553)
Supplement: Table S3 — Rescue of adult flies by UAS-sna overexpression in a twi mutant background. (DOCX) [file pone.0099553.s011.docx]

**Table S3. Rescue of adult flies by *UAS-sna* overexpression in a *twi* mutant background.**

*twi^1^, twi-Gal4/SM6 Cy Roi eve-lacZ X twi^RY50^, UAS-sna/SM6 Cy Roi eve-lacZ*

| **Line** | **Cy** | **Cy^+^** | | **Rescue** |
| --- | --- | --- | --- | --- |
|  |  | Observed | Expected |  |
| 1 | 76 | 42 | 38 | 111% |
| 2 | 29 | 17 | 15 | 113% |

No Cy^+^ flies are ever observed in either parental stock. Two independent crosses were performed with separate *twi^RY50^, UAS-sna/SM6 Cy Roi eve-lacZ* recombinant stocks.
